# Expression estimation and eQTL mapping for HLA genes with a personalized pipeline

**DOI:** 10.1371/journal.pgen.1008091

**Published:** 2019-04-22

**Authors:** Vitor R. C. Aguiar, Jônatas César, Olivier Delaneau, Emmanouil T. Dermitzakis, Diogo Meyer

**Affiliations:** 1 Department of Genetics and Evolutionary Biology, Institute of Biosciences, University of São Paulo, São Paulo, Brazil; 2 Department of Genetic Medicine and Development, University of Geneva Medical School, Geneva, Switzerland; HudsonAlpha Institute for Biotechnology, UNITED STATES

## Abstract

The HLA (Human Leukocyte Antigens) genes are well-documented targets of balancing selection, and variation at these loci is associated with many disease phenotypes. Variation in expression levels also influences disease susceptibility and resistance, but little information exists about the regulation and population-level patterns of expression. This results from the difficulty in mapping short reads originated from these highly polymorphic loci, and in accounting for the existence of several paralogues. We developed a computational pipeline to accurately estimate expression for HLA genes based on RNA-seq, improving both locus-level and allele-level estimates. First, reads are aligned to all known HLA sequences in order to infer HLA genotypes, then quantification of expression is carried out using a personalized index. We use simulations to show that expression estimates obtained in this way are not biased due to divergence from the reference genome. We applied our pipeline to the GEUVADIS dataset, and compared the quantifications to those obtained with reference transcriptome. Although the personalized pipeline recovers more reads, we found that using the reference transcriptome produces estimates similar to the personalized pipeline (*r* ≥ 0.87) with the exception of *HLA-DQA1*. We describe the impact of the HLA-personalized approach on downstream analyses for nine classical HLA loci (*HLA-A*, *HLA-C*, *HLA-B*, *HLA-DRA*, *HLA-DRB1*, *HLA-DQA1*, *HLA-DQB1*, *HLA-DPA1*, *HLA-DPB1*). Although the influence of the HLA-personalized approach is modest for eQTL mapping, the p-values and the causality of the eQTLs obtained are better than when the reference transcriptome is used. We investigate how the eQTLs we identified explain variation in expression among lineages of HLA alleles. Finally, we discuss possible causes underlying differences between expression estimates obtained using RNA-seq, antibody-based approaches and qPCR.

## Author summary

The level at which a gene is expressed can have important influence on the phenotype of an organism, including its predisposition to develop diseases. One way to estimate gene expression is by quantifying the abundance of RNA. RNA-seq has become the method of choice to provide such estimates at the genomewide scale. However, the application of RNA-seq to HLA genes —key players in the immune adaptive response— has remained a rarely explored approach. This is due to the problem of mapping bias, which causes deficient read alignment at genes which are very polymorphic and different from the reference genome. This has motivated approaches that replace the single reference genome with *personalized* sequences, comprised of the individual’s specific HLA genotype. Here we explore the use of computational frameworks to obtain reliable expression levels for HLA genes from RNA-seq datasets. We present a pipeline in which the quantification of HLA expression is carried out using methods which account for HLA diversity, avoiding the biases of standard approaches. We then evaluate the impact of this form of quantifying HLA expression on downstream analyses. The pipeline also allows us to integrate information on eQTLs with expression levels at the HLA allele-level, which can help disentangle different contributions to disease phenotypes and help understand the regulatory architecture at the HLA region.

## Introduction

The HLA region is the most polymorphic in the genome, and also shows the greatest number of disease associations, which has made it very well characterized at the genomic, population and functional levels [1, 2]. Decades of research have also shown that the HLA genes are targets of natural selection, likely a consequence of their role in responding to pathogens [2, 3]. This combination of evolutionary and biomedical interest has resulted in an extensive catalogue of HLA variation in human populations, with the frequency of HLA alleles defined for various populations [4–7].

The most intensely studied genes in the region are the classical HLA loci. These include Class I genes (*HLA-A*, *HLA-C*, and *HLA-B*), whose proteins present endogenous antigens to CD8^+^ T cells, and also regulate innate immune responses by interacting with killer cell immunoglobulin-like receptors (KIR) expressed on natural killer (NK) cells, and Class II genes (*HLA-DRA*, *HLA-DRB1*, *HLA-DQA1*, *HLA-DQB1*, *HLA-DPA1*, and *HLA-DPB1*) whose proteins present exogenous antigens to CD4^+^ T cells [1, 8].

Variation within or near HLA genes has been convincingly linked to resistance and susceptibility to both autoimmune and infectious diseases [2, 9, 10]. Although in many cases the mechanistic basis of the associations remains poorly understood, there has been an effort to identify whether associations can be linked to features such as variation at the level of specific amino-acids, HLA alleles, HLA haplotypes, non-coding variants near HLA genes, or HLA expression levels (reviewed in [2, 10, 11]). In some instances, the association of one feature results from the fact that it tags another feature. Several studies have shown that HLA alleles may have a protective effect because they mark overall gene expression [12–15]. Other studies showed that a combination of two features may be important. For example, mortality of transplant recipients was associated with increased expression of *HLA-C* alleles which harbor specific amino-acids [16].

An understanding of how HLA expression varies among individuals, and the identification of genetic variants involved in the regulation of expression, will play a central role in understanding the contribution of HLA genes to normal and disease phenotypes. However, until recently, little information existed about the regulatory variation and population-level expression patterns of HLA genes, a result of the difficulty in quantifying expression for genes which show an unusually high polymorphism and are members of a multi-gene family [9, 17].

Efforts have been made to develop antibody-based methods to quantify HLA protein on the cell surface [12–14, 18–20], or hybridization-based approaches to quantify mRNA, such as qPCR [13, 21, 22] and microarray methods [23]. However, the design of PCR primers, microarray probes, or antibodies that span the diversity of possible variants represents a technically challenging and labor-intensive undertaking. In addition, qPCR technologies are not appropriate for comparison of expression levels among different loci, an important concern when seeking to understand how expression of HLA genes responds to environmental challenges.

The results obtained to date using qPCR [13, 21, 22], antibody-based approaches [12–14, 18–20] and customized arrays [23] have contributed to the understanding of HLA expression and its underlying genetic regulation. However, they do not take advantage of the large amount of RNA-seq data generated by studies of whole transcriptomes in large samples [24–26], often involving populations from various regions of the world, which represents an attractive resource to investigate HLA expression.

Although such whole-transcriptome RNA-seq studies do provide expression estimates for HLA genes, they bring new challenges. RNA-seq pipelines may provide biased expression estimates for two reasons: 1) many short reads originating from genes with extreme polymorphism fail to map to the reference genome, due to high degree of variation (which results in a large number of mismatches between the reference genome and that of most individuals being analyzed), and 2) the presence of paralogues makes it difficult to map a read uniquely to a specific gene, leading to the exclusion of many reads. This raises concerns about the reliability of RNA-seq approaches to quantify HLA expression, given that these loci represent both the extreme of polymorphism in the human genome and are part of a multi-gene family [27–29].

A strategy to overcome these challenges is the mapping of reads to an HLA-personalized reference (an index containing sequences of the individual being quantified), rather than to a single reference genome. For example, seq2HLA is a tool developed by Boegel et al. [30] to provide *in-silico* HLA types and expression estimates, and later applied to demonstrate that different tumor types are associated with different HLA expression levels [31], and also to provide a large catalog of HLA expression in 56 human tissues and cell types [32]. AltHapAlignR [33] is another software which infers the HLA references which are the closest to the individual’s HLA haplotypes, and maps reads to them. The authors reanalyzed a large RNA-seq dataset [24], and provided comparisons with conventional read mapping, showing an improvement in accuracy with the HLA-tailored pipeline.

In this article we present HLApers, a personalized pipeline which we have developed to reliably quantify HLA expression from RNA-seq data. We compare HLApers to conventional pipelines, and discuss for the first time the impact of accurate estimation of HLA expression on downstream analyses such as eQTL (expression Quantitative Trait Loci) mapping and allele-specific expression. We show that it is possible to adapt different computationally efficient methods to work under the personalized reference framework, providing reliable quantification of HLA expression from RNA-seq data. We find that implementations with either a conventional read mapper [34] or a pseudoaligner [35] show similar expression estimates.

We use simulations to assess accuracy, showing that the HLA-personalized pipeline is more accurate than conventional mapping, and apply the tool to reanalyze RNA-seq data from the GEUVADIS Consortium [24], which made available whole-transcriptome RNA-seq data for Lymphoblastoid Cell Lines (LCLs) from 462 European and African individuals. We evaluate the impact of more accurate expression estimates obtained with HLApers on downstream analyses by carrying out a detailed survey for allele-specific expression and eQTL mapping at the classical HLA loci.

Surprisingly, we find that conventional RNA-seq pipelines provide gene-level expression estimates and identify eQTLs which are highly correlated with those obtained under the HLA-personalized approach. However, we identify gains of using a pipeline tailored for HLA expression: higher accuracy, expression estimates at the HLA allele-level, and eQTLs with higher probabilities of being causal.

## Results

### Estimating HLA expression with HLApers

**Fig 1:**
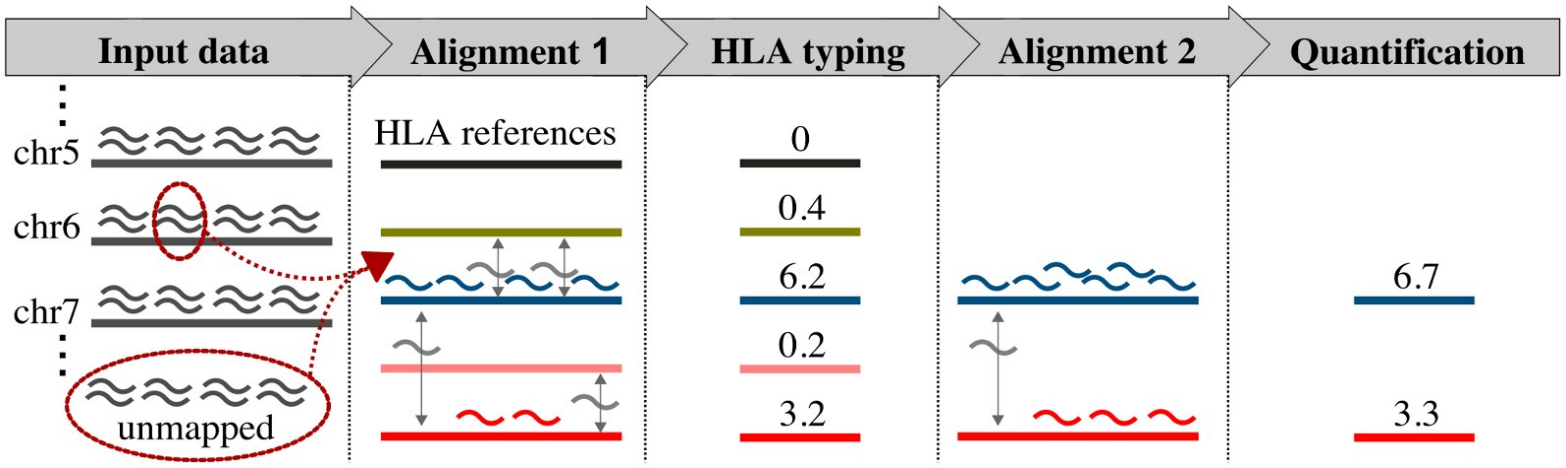
Schematic representation of the HLApers pipeline to estimate HLA expression. The reads generated by RNA-seq are represented by curved shapes. The pipeline can use as input either reads in fastq format or extracted from a BAM file (*Input data*). These reads are aligned to an index containing references for all known HLA alleles (*Alignment 1*). We next identify the references to which most reads align, so as to infer the HLA genotypes of the individual at that locus, in this example a heterozygote carrying a blue and a red allele (*HLA typing*). Next, a second alignment is performed, using as an index only the references for the alleles inferred to be present in that individual at that locus (*Alignment 2*). Expression is estimated based on the number of reads aligning to each reference, using a statistical model that accounts for instances where reads align to multiple HLA references corresponding to different HLA alleles or genes (*Quantification*). For each HLA allele, the quantification corresponds to the proportion of the total reads assigned by the statistical model.

We developed the HLApers pipeline (for HLA expression with personalized genotypes) to measure HLA expression from whole-transcriptome RNA-seq data. The pipeline can use either (1) a suffix array-based read mapper (STAR [34]) followed by quantification with Salmon [36] (henceforth called STAR-Salmon), or (2) a pseudoaligner with built-in quantification protocol (kallisto [35]). The key feature of our implementation is the use of an index supplemented with a set of sequences covering the breadth of known HLA sequences (see Materials and methods: Index supplemented with the HLA diversity). We implemented a two-step quantification approach, where (1) we align reads to reference sequences corresponding to all known HLA alleles, and identify those which maximize the read counts at each locus to infer the genotype which is present (*in-silico* genotyping), and (2) we use this inferred HLA genotype to create a personalized index which we use to quantify expression (Fig 1).

A problem for the analysis of HLA is that of multimaps: reads originating from a particular allele which map to other alleles of the same locus, or to other loci. To deal with multimaps different approaches have been used. One possibility is to discard them (as in [33]), and another is to split them evenly among the compatible references (as in [30]). In HLApers, we use maximum likelihood estimates of expression obtained by an expectation-maximization (EM) algorithm (implemented within Salmon [36] and kallisto [35]), which infers the quantities of each HLA allele that maximize the probability of observing the set of sequenced reads.

Because the *in-silico* typing is an important step for accurate expression estimates, we assessed the concordance between our RNA-seq based HLA typing and the HLA allele calls experimentally determined for 5 HLA loci using Sanger sequencing [6]. The concordance was higher than 97% for all of the HLA genes compared (S1 Table). This is consistent with previous results showing that RNA-seq provides reliable HLA alleles calls [30, 37–40].

Our HLApers pipeline integrates widely used quantification tools such as Salmon and kallisto, and provides both allele and locus level estimates of HLA expression. The pipeline is available at https://github.com/genevol-usp/HLApers.

### HLApers performance on simulated data

We next investigated how using the HLA-personalized index affects the quantifications of HLA expression. To this end we simulated an RNA-seq experiment using the Polyester package [41], with a dataset of 50 individuals, with the read length and counts matching those of the observed data (see Materials and methods: Simulation).

We analyzed the simulated dataset with three different methodologies: (1) the two-step approach in HLApers, where we first inferred the personalized HLA genotype and then aligned reads to it; (2) alignment to the reference transcriptome (Gencode release 25; primary assembly); and (3) alignment to the reference genome (GRCh38). In approaches (1) and (2) the alignment is followed by expression quantification using Maximum Likelihood (ML), which provides a statistical framework for dealing with multimap reads, whereas in approach (3) quantification is performed using only uniquely mapped reads.

**Fig 2:**
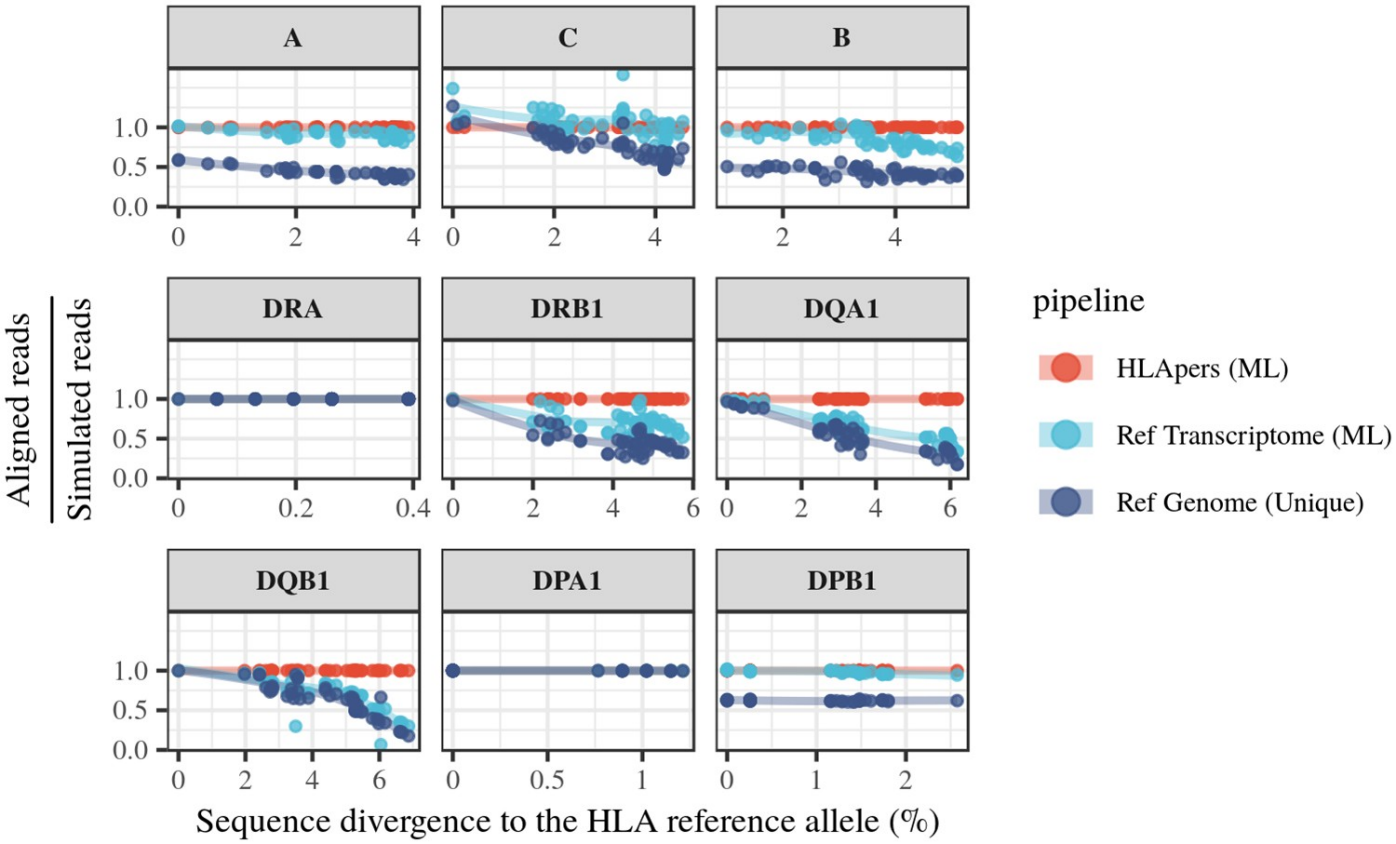
Alignment success with three different methodologies. The proportion of simulated reads which successfully aligned is defined for each locus by the ratio of the estimated read counts to the number of simulated reads (y-axis). These values are displayed as a function of the divergence of the HLA allele to the reference genome (x-axis). Simulated reads were processed through 3 different pipelines: (1) alignment to the personalized HLA sequences (*HLApers (ML)*), (2) alignment to the reference transcriptome (*Ref Transcriptome (ML)*), (3) alignment to the reference genome considering only uniquely mapped reads (*Ref Genome (Unique)*).

For each HLA locus and methodology, we assessed the proportion of simulated reads which successfully aligned. Because previous studies for genome sequencing identified a correlation between mapping success and the number of mismatches between the HLA allele an individual carries and the reference genome [28], we analyzed how alignment success behaves as a function of the number of mismatches between each HLA allele and the reference genome (Fig 2).

The use of an HLA-personalized index results in the largest proportion of successfully aligned reads, no matter how different the allele carried by the individual is from the allele in the reference genome. This is expected, since the personalized HLA component guarantees that a sequence close or identical to that originating the read will be present.

When the alignment was performed using the reference transcriptome, there was a marked reduction in the proportion of successfully aligned reads for *HLA-DRB1*, *HLA-DQA1*, *HLA-DQB1*, driven by decreased alignment success for alleles with a greater proportion of mismatches with respect to the reference genome.

When using uniquely mapped reads there was a massive read loss for *HLA-A*, *HLA-B* and *HLA-DPB1*, regardless of the divergence to the reference genome, as well as a lower proportion of successfully aligned reads across other loci. This shows that both discarding multipmaps, as well as not including a personalized index, have a negative impact on mapping success.

Finally, for the least polymorphic HLA loci, mapping should not be sensitive to the specific reference used. This is precisely what we find, with all pipelines performing similarly for *HLA-DRA* and *HLA-DPA1*.

### HLApers analysis of the GEUVADIS dataset

**Fig 3:**
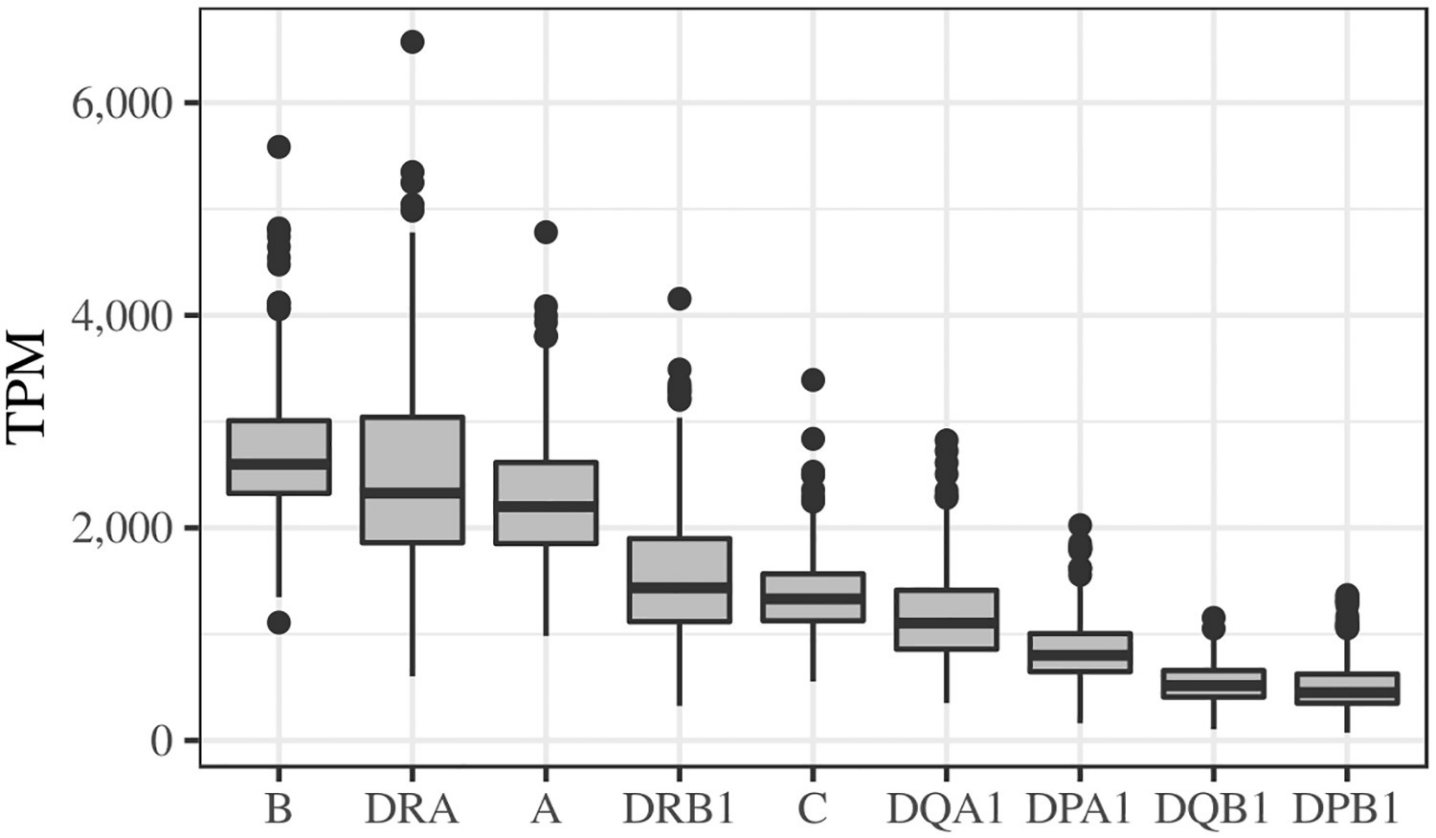
Gene-level expression of classical HLA genes in 358 European individuals from GEUVADIS [24]. Expression was estimated with the HLApers pipeline. Horizontal lines inside each box represent the median. The lower and upper hinges correspond to the first and third quartiles respectively. The whisker lines extend from the hinges to the largest value no further than ±1.5 × *IQR* from the hinge (where IQR is the inter-quartile range, or distance between the first and third quartiles). Data beyond the end of the whiskers (“outliers”) are plotted individually. TPM: Transcripts per Million.

**Fig 4:**
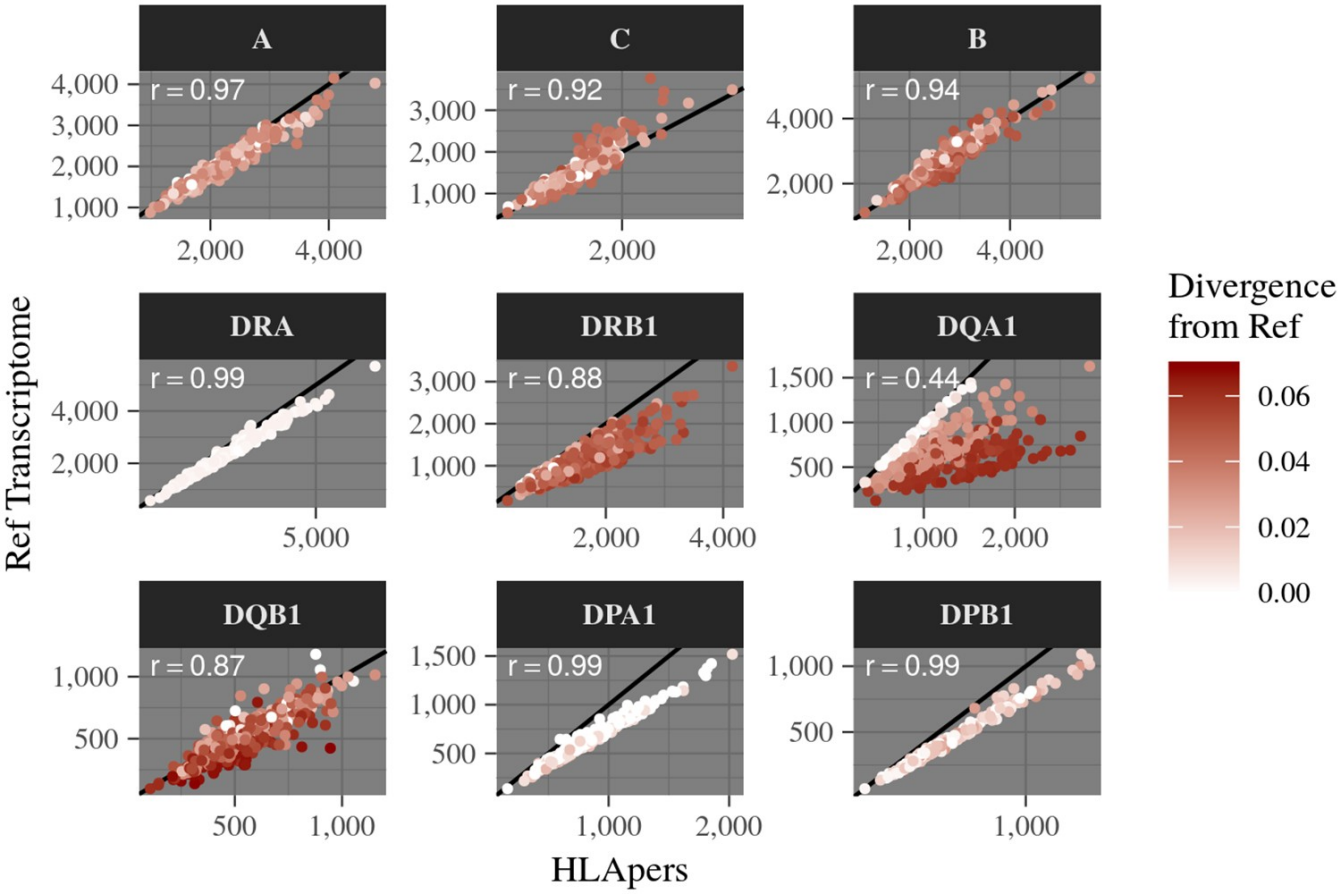
Expression estimates using personalized HLA genotypes (x-axis) versus the reference transcriptome (y-axis) in the index. For each individual we identify the average divergence (proportion of mismatches) with respect to the reference genome, indicated by the red gradient. For all loci with low correlations between expression quantification methods, there is a marked drop in expression estimates using the reference transcriptome when the alleles are most divergent from the reference. Expression estimates are in Transcripts per Million (TPM). r: Pearson correlation coefficient.

Having demonstrated that including an individual’s HLA alleles in the index improves the success of read alignment in the simulated data (Fig 2), we set out to address two questions with real data by applying HLApers to the GEUVADIS dataset [24]. First, we examined how expression varies among HLA loci, when the personalized index is used (Fig 3). Secondly, we compared expression estimates with and without the use of the personalized index, so as to evaluate the impact of its usage on real data (Fig 4).

By summing the estimates for the 2 alleles at each HLA locus, we obtain gene-level expression estimates (Fig 3). We observe that *HLA-B* is the highest expressed gene overall. Among the Class I genes, *HLA-B* is followed by *HLA-A* with similar levels, and by *HLA-C* which has about 50% of the expression levels of *HLA-B*. For Class II genes, *HLA-DRA* is the most highly expressed. Although we observe a general concordance with the original GEUVADIS quantifications [24], there are some notable differences: in the original quantifications, *HLA-B* is twice as expressed as *HLA-A*, and *HLA-DPA1* is more expressed than *HLA-DRB1* (S1 Fig).

We found that the correlation between results using the reference transcriptome or the personalized index was greater than 0.87 for every locus except for *HLA-DQA1* (Fig 4). The loci with the lowest correlations between indices (*HLA-DRB1*, *HLA-DQA1* and *HLA-DQB1*), are also those with the greatest read loss when divergence from the reference allele is high in the simulation (Fig 2).

We then investigated if the quantification tool used in HLApers (STAR-Salmon or kallisto) influences expression estimates. Correlations between STAR-Salmon and kallisto approaches were on average *r* > 0.99 for read counts, dropping to *r* = 0.8 for TPM estimates for Class I genes, likely due to different bias correction models (S2 Fig). These results show that the key features influencing alignment success are the use of a personalized index and the statistical treatment of multimaps (as opposed to discarding them), with the specific alignment tool being less influential.

Finally, we evaluated the reproducibility of the HLA expression estimates obtained with HLApers. To this end, we analyzed replicates for 97 European individuals in GEUVADIS. We observed an average correlation of expression estimates between replicates of 0.92 over the 9 classical HLA loci. This shows that HLA quantifications from RNA-seq with HLApers are highly reproducible (S3 Fig).

### eQTL analysis of HLA loci

The key role played by HLA loci in the immune response and their strong and abundant associations with infectious and autoimmune diseases have motivated studies to uncover their regulatory architecture [15, 20, 21, 23, 42–45].

Here we use accurate expression estimates obtained with HLApers, together with genotype data from the 1000 Genomes Project [46], to identify SNPs which are associated with variation in expression levels (eQTLs).

**Fig 5:**
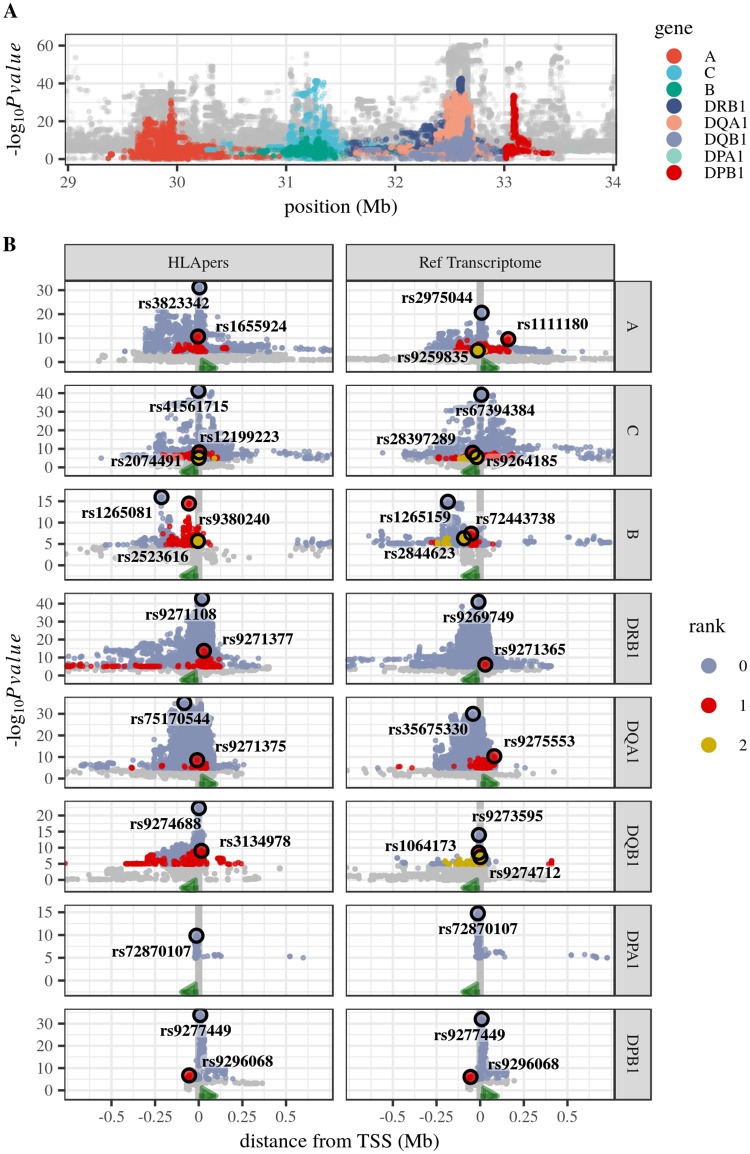
eQTLs for HLA loci. A: Distribution of p-values for the association between genotypic and expression variation along the HLA region, colored by gene. B: Distribution of p-values around the transcription start site (TSS, vertical line at x = 0), for eQTLs mapped with HLApers or reference transcriptome pipelines. Points are colored according to the rank of association (with gray for non-significant associations at an FDR of 5%). The best eQTL of each rank is circled in black. Green arrows indicate the orientation of transcription. There was no eQTL mapped for *HLA-DRA*.

Because multiple SNPs can affect expression, it is interesting to identify independent contributions made by distinct SNPs. This has previously been done by using a best eQTL (i.e., the one with the most extreme p-value) as a covariate in subsequent searches for an additional variant. Here we use a related approach implemented in QTLtools, a collection of tools to perform eQTL analysis [47]. The module QTLtools cis allows for the identification of groups (or “ranks”) of SNPs associated with independent signals. For each rank, we identified the site with the most extreme association (Fig 5).

#### eQTL differences across indices

Although expression estimates obtained using HLApers or reference transcriptome are significantly correlated, for some loci bias affects estimates from the reference transcriptome for HLA alleles which are highly diverged from the reference (Figs 2 and 4). We therefore examined if these differences in expression across indices have a direct effect on the eQTLs identified.

Even when eQTLs across studies or pipelines are different, they may tag the same biological signal (possibly driven by the same causal site). A high level of linkage disequilibrium (LD) between two SNPs is not enough to claim shared biological signal, since the LD structure in the genome may produce coincidental overlaps of variants which are unrelated in respect to a given regulatory function [48]. However, if two SNPs tag the same signal, we expect to see a decrease in the strength of association of one SNP when removing the effect of the second SNP. This is the logic underlying the Regulatory Trait Concordance (RTC) method [47, 48], which we use to test if eQTLs from different pipelines mark the same signal. An RTC score close to 1 indicates that a pair of SNPs likely capture the same biological signal.

We found that the lead eQTLs capture the same biological signals across indices for all loci except *HLA-DRB1* (S2 Table). Despite this sharing of eQTL discoveries, the p-values for the eQTLs mapped under the HLA-personalized pipeline are almost always more extreme than those from the reference transcriptome (Fig 5).

#### eQTL sharing with previous studies

Next we investigated whether the eQTLs which we mapped share the same causal signal as those reported by previous studies. We first queried selected publications which focused on expression of genes with immune function, and that described SNPs with a functional role on HLA expression (in some cases with experimental validation) [12, 13, 42, 45, 49–51].

We found that our eQTLs for *HLA-C* are very highly correlated at the haplotypic level (0.95 ≤ *D*′ ≤ 1) with the variants described by Thomas et al. [12] (a -35bp SNP in *HLA-C* promoter), Kulkarni et al. [42] (a SNP which regulates *HLA-C* expression via a 3’UTR miRNA binding site), Fairfax et al. [49] (a genome-wide significant eQTL in PBMC (Peripheral Blood Mononuclear Cells)), and Vince et al. [50] (a SNP in the Oct1 binding site in the *HLA-C* promoter region). A similar pattern holds for the rank 1 eQTL which we mapped for *HLA-DQB1*, which is in high LD (*D*′ = 1) with a variant reported by Raj et al. [45] within a super-enhancer which regulates *HLA-DQA1*, *HLA-DQB1* and *HLA-DRB1*. For HLA-DP we also found strong association (*D*′ = 1) between our eQTLs and previous SNPs with regulatory role for *HLA-DPB1* [13] and *HLA-DPA1* [51] (S3 Table).

However, as we discussed above, the RTC method is more appropriate than linkage disequilibrium (LD) to evaluate the sharing of causal signals. When applied to these SNPs which are in high LD with our eQTLs, we find that only the association between our *HLA-C* rank 1 eQTL and previously identified regulatory SNPs was significant according to the RTC criterion, showing a high probability (*RTC* = 0.99) that our eQTL shares the same biological signal with the SNP of Fairfax et al. [49]. We next expanded our RTC analysis to a catalog of previously described SNPs, including large eQTL studies of LCLs [24, 26, 52]. We find that, overall, 9 of the 17 eQTLs we identified likely share the same causal signal as previous eQTLs or experimentally validated SNPs (S3 Table).

Remarkably, only 1 of the 6 eQTLs mapped for *HLA-DQA1*, *HLA-DQB1*, and *HLA-DRB1* share a signal with previous eQTLs. This is consistent with our finding that for these genes the expression estimates are affected by divergence from the reference genome (Figs 2 and 4), which may have led to incorrect estimation of expression in previous studies.

#### Putative function of eQTLs

To gain insight into possible function of the eQTLs mapped with HLApers, we examined their location with respect to the associated HLA gene and functional annotation for LCLs in The Encyclopedia of DNA Elements (ENCODE [53]) (S4 Table).

Considering all ranks of association, we mapped 17 eQTLs for a total of 8 classical HLA genes. *HLA-DRA* is the least polymorphic classical gene, and had no associated eQTL. The lead eQTL was located no further than 20kb from the gene for all classical HLA genes except *HLA-DQA1*, for which the lead eQTL was 82kb upstream the transcription start site (TSS), but within a cloud of associations which extends to the TSS, and for *HLA-B* where the lead eQTL was 210kb away, an unusually large distance with respect to that of the other loci, but still within a range found in the genomewide eQTL data (where 11.1% of the eQTLs lie at that distance or further).

Six of the 17 eQTLs are part of TFBS (Transcription Factor Binding Sites) or DHS (Dnase I Hypersensivity Sites). Regarding chromatin modification, 10 eQTLs were located within regions with marks of active promoters, enhancers or other distal elements, transcribed genes, or elements with dynamic chromatin (S4 Table).

We also used RTC to evaluate if eQTLs identified in our study capture the same signals as CRD-QTLs identified by Delaneau et al. [52]. CRDs (for Cis-Regulatory Domains) are segments of the genome defined by chromatin activity and formed by coordination between nearby regulatory elements [52] (see S4 Fig for the location of HLA genes with respect to CRDs in the region). A high RTC score between a CRD-QTL and an eQTL indicates a shared influence on the formation of a CRD and on gene expression. Overall, 9 out of 17 eQTLs we identified mark the same signal as CRD-QTLs. Interestingly, the lead eQTL for *HLA-B*, which is 210kb away from the gene, has an RTC score of 0.99 with a variant associated with the activity of the CRD linked to *HLA-B* (S5 Table).

Studies have revealed a significant overlap of eQTLs and variants identified in GWAS (Genome-Wide Association Studies), which provides insight into a biological basis for the GWAS association [11, 24, 54–56]. Most GWAS variants are non-coding, and in those cases it is difficult to know which variant is causal and which gene is modulated, complicating the identification of the direct effects of variants on disease etiology [48, 57]. The mapping of eQTLs, by identifying variants involved in modulating expression, offers an additional layer of information for interpreting GWAS hits. Thus, we sought to investigate the overlap between eQTLs we mapped and GWAS signals.

We found that nearly all of our eQTLs, according to the RTC score, likely share the same biological signal as GWAS hits (S6 Table), which shows an extensive implication of regulation of gene expression in disease phenotypes at the HLA region. However, the high density of GWAS hits in the HLA region, coupled with a high linkage disequilibrium, provides an alternative explanation for this pattern.

#### Causality

A key challenge for eQTL studies is identifying which variants are causally related to variation in expression, and which are simply genetically correlated with the functional variant. One approach to identify causal variants is to use empirical and simulated data on eQTLs in a probabilistic framework, to estimate the probability that a variant is causal. We used this approach, as implemented in CaVEMaN software [55], to estimate the probability that the eQTLs we mapped are causal, and to evaluate if more accurate expression estimates could lead to improved mapping of causal variants.

For 10 out of 14 eQTLs (excluding HLA-DP, for which eQTLs are the same across pipelines), we found that the HLApers produces eQTLs with higher probabilities than the reference pipeline. However, most variants do not reach probabilities of 0.8, indicating that even though there is an increase in probability of causality when using HLApers, larger samples sizes are important to improve fine mapping (S5 Fig).

### HLA allele-level analysis

Transcriptome studies can quantify expression for various biological features: individual SNPs, exons, isoforms, genes. In the case of HLA loci, a natural unit of interest is the HLA allele. HLApers provides expression estimates for individual alleles, since it is the allelic sequences which are included in the index. The immunogenetics literature has shown that many HLA alleles are associated with specific phenotypes of evolutionary and medical importance (reviewed in [2, 10, 11]). Gauging information about the expression levels of alleles can therefore provide an additional layer of information.

In order to explore allele-level estimates, we first grouped alleles in “lineages”, which comprise groups of alleles which are evolutionarily and functionally related, since the large number of alleles would make sample sizes per allele too sparse. Although the expression of individual allelic lineages is highly variable among individuals, there is an overall significant difference in expression among lineages (Welch’s ANOVA p-values ranging from 3.7 × 10^−8^ for *HLA-DPB1* to 6 × 10^−51^ for *HLA-DQA1*).

#### Concordance with previous allele-level estimates

We then compared our allele-level expression with those previously obtained for *HLA-A*, *HLA-B* and *HLA-C* by qPCR [21, 22, 43]. For each locus, we restricted our analyses to lineages in at least 10 individuals, and with significantly different expression from at least one other lineage (Mann-Whitney U test with FDR correction for multiple testing, *p* < 0.05). We found 33 instances where the expression was significantly different between a pair of lineages in both qPCR and RNA-seq datasets, and in 21/33 cases the results were concordant (i.e., we found the same ordering of expression in RNA-seq and qPCR). However, the results were markedly different among loci: for *HLA-C*, 13 out of 13 significant pairs were concordant, for *HLA-B*, 4 out 6 were concordant. On the other hand, for *HLA-A*, qPCR and RNA-seq results were concordant in only 4 out 14 pairs (see S1 Text for details). Future scrutiny will be needed to determine whether qPCR and RNA-seq have biases specific to *HLA-A*, or if these differences can be explained by biological factors such as different populations/samples and tissues/cell types.

#### HLA allele lineages and eQTLs

To investigate HLA regulation at the allele-level, we estimated expression levels for HLA alleles, and we inferred haplotypes that spanned HLA loci and eQTLs, so that the phasing between these was known (see Materials and methods: Phasing of HLA alleles).

**Fig 6:**
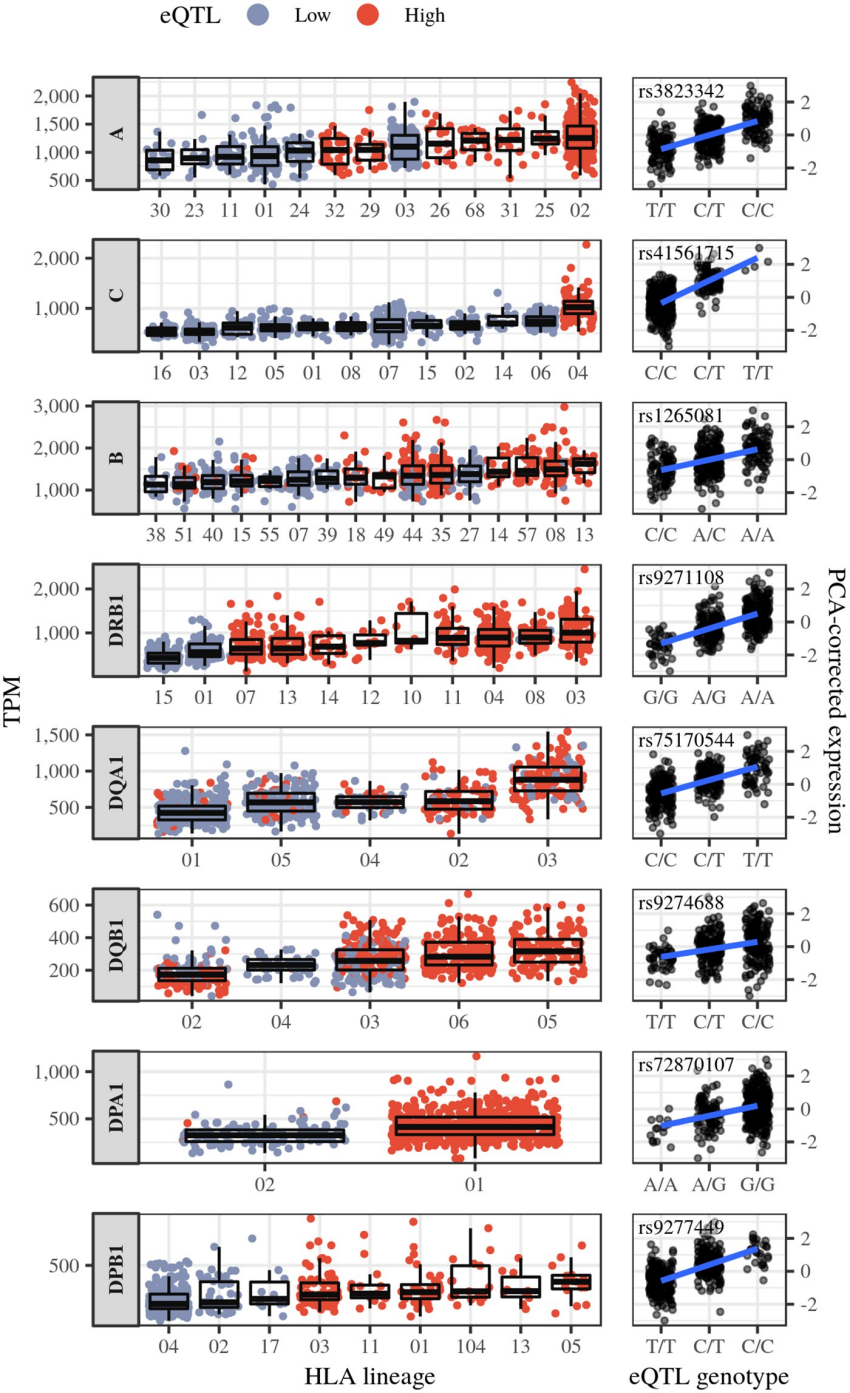
Expression levels for different HLA lineages and eQTL genotypes. For each locus panel we have: (left-hand side) expression estimates for each HLA allele in Transcripts per Million (TPM), and (right-hand side) gene-level estimates for each eQTL genotype in PCA-normalized estimates used for eQTL mapping. Allelic lineages with ≥ 10 individuals are shown.

We investigated the degree to which the mapped eQTLs account for variation in expression levels of HLA alleles. Frequently, we observe that more highly expressed HLA alleles are on haplotypes carrying the eQTL allele associated with increased gene-level expression (red dots in Fig 6). At *HLA-C*, for example, the rs41561715-T allele is exclusively associated with the C*04 lineage. Another eQTL completely linked to HLA lineages is seen at *HLA-DPB1*. The alleles at this locus were previously divided into two clades, marked by the variant rs9277534 in the 3’-UTR [13, 58, 59]. The alleles DPB1*02, DPB1*04 and DPB1*17 are on the same haplotype as rs9277534-A, and have low expression. The other alleles are on haplotypes carrying rs9277534-G, and have higher expression [13, 58, 59], which may increase the risk of persistent Hepatitis B virus (HBV) infection [13]. Here we mapped rs9277449 as the lead eQTL for *HLA-DPB1*. We found that rs9277449 and rs9277534 are non-independent (2kb apart, *D*′ = 1, both contained among the rank 0 variants (blue) in Fig 5), and rs9277449 also separates the two clades of alleles based on expression (Fig 6).

These results show that reliable eQTL mapping may help explain differences in expression among HLA alleles, and help identify genetic variants which contribute to disease risk.

#### Allelic imbalance

There is increasing interest in understanding if gene expression is unbalanced (i.e., one allele is more expressed than the other), and if so, how this contributes to interpretations of disease phenotypes and natural selection [60]. In the case of HLA genes, for which heterozygote advantage is a selective regime with strong theoretical support, extremely unbalanced expression poses a theoretical challenge, since being heterozyote would not be advantageous if only one allele is expressed. We have used our expression estimates to quantify allelic imbalance for HLA genes.

**Fig 7:**
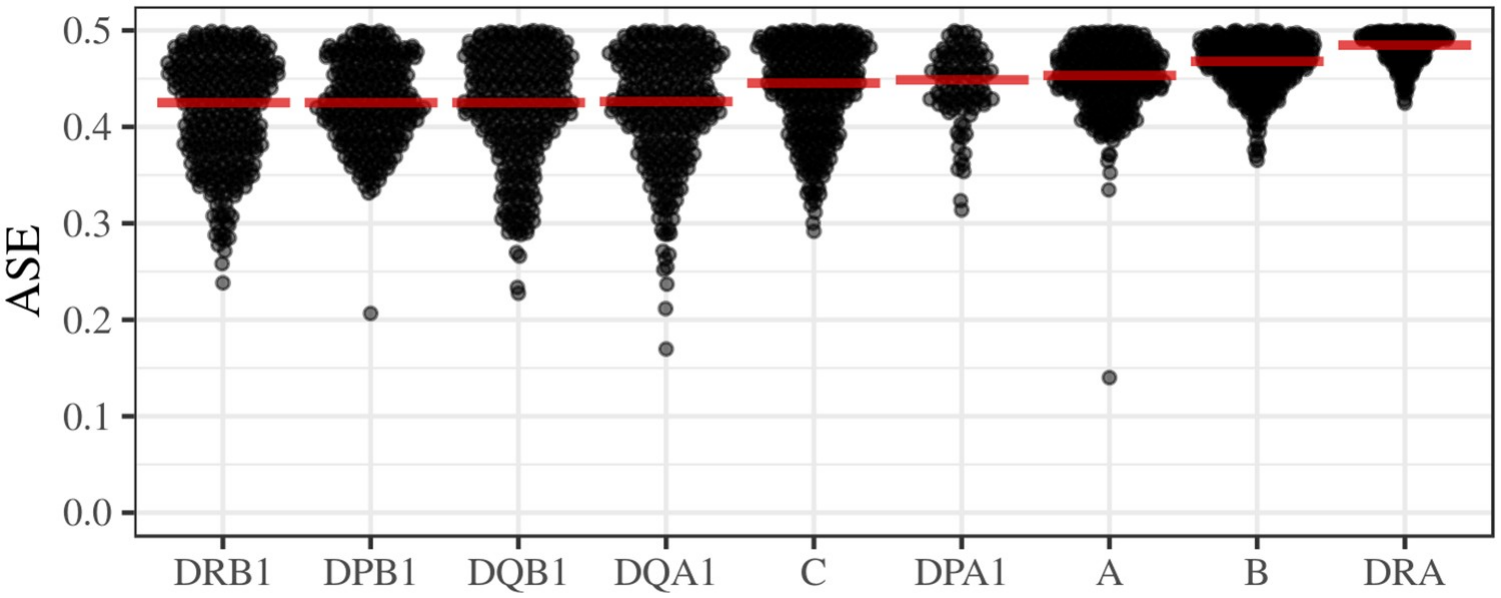
Allele-specific expression. ASE was measured as the proportion of the locus-level expression attributed to the less expressed HLA allele in heterozygous genotypes. Red horizontal bars indicate the median.

Using the expression data at the HLA allele-level we found low asymmetry in expression (Fig 7), with 81% of the heterozygotes having ASE (Allele-Specific Expression) between 0.4 and 0.5 (with Class II genes showing a distribution with a larger variance), and no instances of extreme imbalance as recently reported for SNPs in HLA genes [61]. Therefore, the extreme imbalance seen at the SNP level does not hold at the HLA allele resolution.

### Co-expression and haplotypic coordination in expression

**Fig 8:**
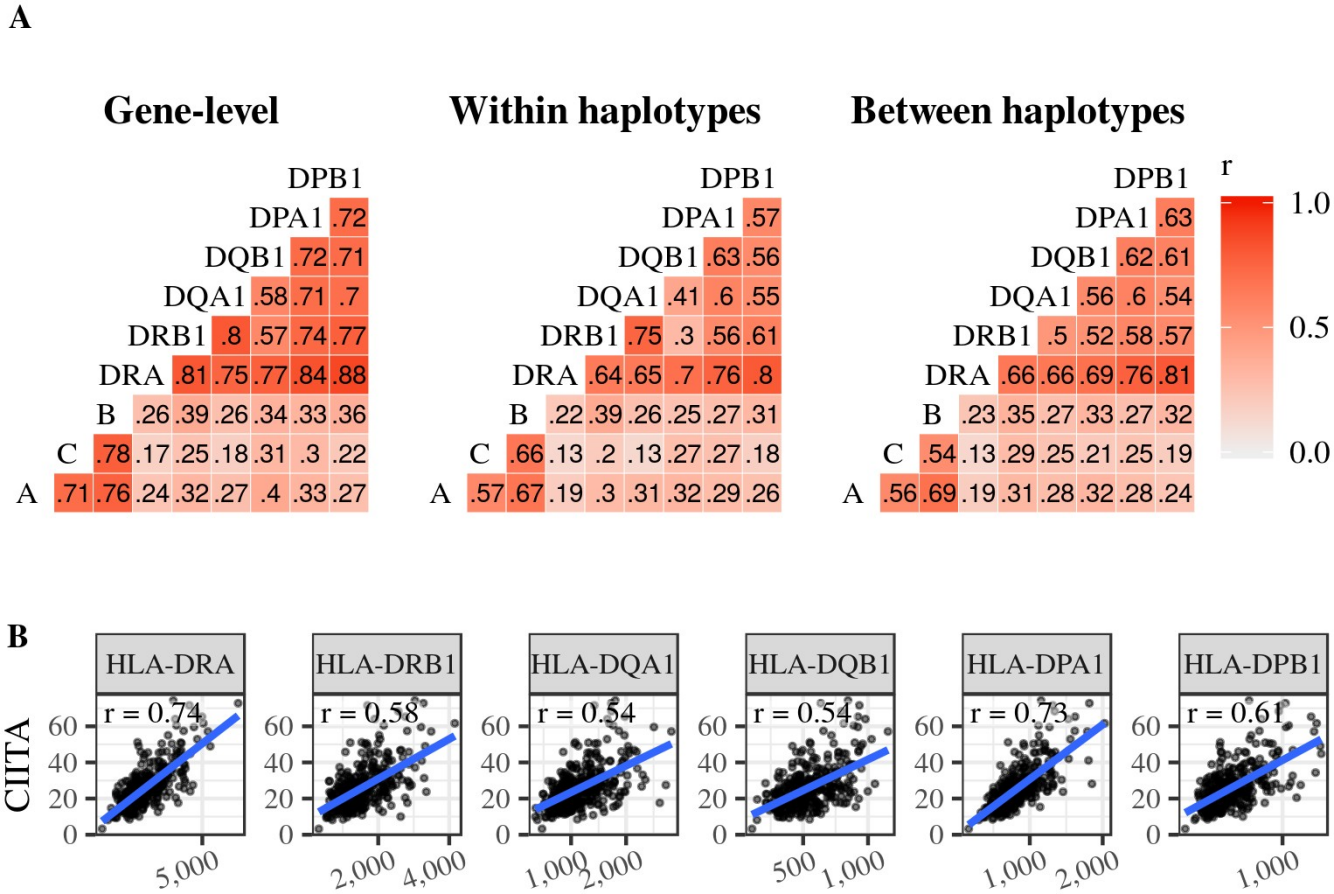
Co-expression patterns for HLA genes. A: Pairwise correlation of expression estimates at the gene-level, for alleles from within the same haplotype, and for alleles on different haplotypes. B: An example of a factor that contributes to correlation of expression at the gene level. *CIITA* is located on chromosome 16, and its protein regulates the expression of Class II genes. r: Pearson correlation coefficient.

Enhanced coordination of gene expression has been proposed as an advantage for gene clustering, as seen at the HLA region [1, 2]. We found a high correlation of expression both within the group of Class I and Class II genes, and lower levels between Class I and Class II genes, which are more than 1Mb apart (Fig 8A) (but see [22] for a result of no co-expression among Class I genes).

A possible cause for co-expression of genes which are physically close to each other is that regulatory activity is structured in domains (CRDs, for Cis Regulatory Domains) [52]. Such domains comprise contiguous regions along a chromosome, and their existence predicts co-expression along haplotypes for genes associated with the same CRD. In fact, previous studies did find evidence that expression of some genes in the HLA region was a feature associated with haplotype membership [23, 62].

We used our inferences of HLA haplotype structure to investigate if there is an haplotypic effect on coordination of expression among nearby HLA genes. Specifically, we tested the hypothesis that co-expression is stronger between alleles located within the same haplotype than between those on different haplotypes. We did not find a consistently higher correlation of expression for alleles on the same haplotype (correlation within haplotypes being higher in only 7 out of 18 locus pairs surveyed within Class I or Class II, Fig 8A).

This result suggests that correlation of expression among HLA loci in LCLs is a result of factors acting at the gene level, and is not driven predominantly by properties of the haplotype. For example, we observe correlation of expression between Class II genes and *CIITA*, the Class II Major Histocompatibility Complex Transactivator. This correlation is not driven by proximity (*CIITA* is on chromosome 16), but rather by a *trans* regulatory mechanism (Fig 8B).

## Discussion

The contribution of HLA variation to normal and disease phenotypes goes beyond peptide specificity, and includes other factors which influence the strength of immune responses, such as HLA expression levels. However, we are still only starting to understand the regulation of expression of these genes, a consequence of their extreme polymorphism which imposes challenges to the methodologies available to measure mRNA and protein levels.

There has been an increasing effort to use RNA-seq data for *in-silico* HLA typing ([63] and other methods reviewed in [64]) and to estimate expression levels for HLA genes [30, 33]. In this paper, we present HLApers, an HLA-personalized pipeline that quantifies HLA expression based on RNA-seq. We used these expression estimates to identify eQTLs for HLA genes, to estimate the degree of allelic imbalance in expression, and to investigate how the alleles at eQTLs explain variation in expression among HLA alleles.

When new technologies/methods are developed, it is a good practice to evaluate if the results are in agreement with the current knowledge, which in the case of HLA was mostly derived from qPCR or antibody-based studies. However, in our survey of this literature, we found it difficult to compare our newly obtained expression estimates based on RNA-seq with those from previous studies. There are several reasons underlying this difficulty, which we now discuss.

First, expression estimates based on qPCR and antibody-based approaches are in general not comparable among different HLA loci, since different primers/antibodies are often developed for each locus. Thus, the information for expression differences which we observe among HLA loci are usually not available from studies using these techniques. A few studies developed antibodies with comparable affinities for HLA-A/B/C, allowing comparison of expression between loci. Using such a method, Apps et al. [19] found an at least 12-fold reduction in HLA-C expression with respect to HLA-A and HLA-B as measured by flow cytometry, whereas the difference we documented based on RNA-seq was only twofold. However, the Apps et al. [19] study was restricted to a specific haplotype, and because our results show substantial within locus variation in expression levels, it is difficult to compare studies.

Second, many additional layers of biological factors make a direct comparison across studies challenging. For example, the abundance of total protein, of proteins on cell surface or of mRNA in the cell, represent different molecular phenotypes associated with gene expression, given the existence of various post-transcriptional and post-translational regulatory processes [18, 20, 42, 43, 65, 66]. As a consequence, there are good biological reasons to expect differences when expression is estimated at each of these levels. In addition, it is well known that gene expression varies among tissues and cell types [26, 32], posing an additional challenge in comparisons between our findings and those previously reported in the literature, which are often based on analyses of different cell types or tissues.

Third, regarding mRNA quantification, qPCR and RNA-seq each have their own sources of bias, and the finding that there are differences between studies using these approaches should not, on its own, provide definitive evidence that one or the other is inaccurate. Future studies will need to evaluate the degree to which these two technologies can be compared for quantification of HLA expression.

Nevertheless, given the availability of qPCR based quantifications for HLA expression, as well as the interest in obtaining allele-level estimates of expression, we compared the expression for individual HLA lineages obtained by RNA-seq with those from qPCR from previous studies. We found that RNA-seq and qPCR show high concordance for the relative ordering of lineage-level expression for *HLA-B* and *HLA-C* (66% and 100%, respectively), whereas for *HLA-A* concordance was low, at 28%. Therefore results suggest that for *HLA-B* and *HLA-C*, when we examine lineages that are sufficiently different in their expression relative to one another (see S1 Text), the two methods are in high agreement, but further research is required to identify the sources of discordance between RNA-seq and qPCR for *HLA-A*. It is important to recall that these analyses rely on lineage-level expression estimates obtained by qPCR which are indirect, since they estimate locus-level expression (whereas RNA-seq data analysis with HLApers distinguishes among alleles), adding another source of differences among methods.

Our perspective is that differences across studies call for further investigation, and highlight the potential biological variation that needs to be accounted for when comparing cell types, infection status, methodological approach, and molecule being quantified.

In this study we present HLApers, and use simulations and empirical data analyses to show that it produces estimates for HLA expression from RNA-seq with high precision and accuracy. HLApers incorporates a Maximum-Likelihood estimator to deal with instances of reads mapping to multiple alleles or loci, following a strategy that has been widely used in RNA-seq studies [26, 67]. Our pipeline can use different alignment strategies (e.g. STAR-Salmon [34, 36] or kallisto [35]), but we show that the accuracy of expression depends on the sequences contained in the index, and is less dependent on the program used.

The impact of using an HLA-personalized index on expression estimates varies markedly among loci. We found that for *HLA-DQA1* there are large differences between gene-level expression estimates obtained using the reference transcriptome and those obtained using the HLA-personalized index. However, this difference is quite low for Class I genes, and intermediate for Class II loci other than *HLA-DQA1* (Figs 2 and 4). However, even for the Class I genes, using the personalized index does result in changes in expression estimates with respect to the reference transcriptome. Therefore, we asked whether these changes have an impact on downstream analyses, such as eQTL mapping.

When we compare the eQTLs identified using either HLApers or the reference transcriptome, 6 out of 8 loci had non-overlapping eQTLs. However, in most cases the same biological signal was being captured (S2 Table), and the p-values (Fig 5) and the causality (S5 Fig) of the eQTLs obtained with HLApers are only modestly better than when the reference transcriptome is used. We show that most of the eQTLs we identified (14/17) share a signal with previously reported regulatory SNPs (either experimentally validated SNPs and eQTLs (S3 Table) or CRD-QTLs (S5 Table)). This indicates that, despite of the improvement in expression estimates that personalized pipelines can generate, larger sample sizes are necessary in order to identify eQTLs with greater probability of being causal, and to identify novel eQTLs with smaller effects. We note that the use of transformed cell lines (such as LCLs) may lead to expression profiles specific to this environment, and as a consequence some of the regulatory architecture underlying the expression of the HLA genes may not be shared with other tissues, cell types or treatments.

The HLA-personalized approach provides expression estimates at the HLA allele level, which is not a product of standard RNA-seq pipelines. We integrate allele-level information with the eQTLs mapped for the genes, showing that the HLA allele is a relevant layer of information to understand the regulation of gene expression, because in some instances the regulatory architecture is linked to specific HLA alleles. This joint mapping of regulatory variants and assessment of expression of HLA alleles can illuminate the understanding of the HLA regulation, and contribute to disentangle specific contributions to disease phenotypes.

## Materials and methods

### Ethics statement

All data were obtained from third party sources and no additional ethical approval was required.

### Index supplemented with the HLA diversity

In order to create the index, we downloaded 16,187 nucleotide sequences for 22 HLA loci (*HLA-A*, *HLA-B*, *HLA-C*, *HLA-E*, *HLA-F*, *HLA-G*, *HLA-DMA*, *HLA-DMB*, *HLA-DOA*, *HLA-DOB*, *HLA-DPA1*, *HLA-DPB1*, *HLA-DQA1*, *HLA-DQB1*, *HLA-DRA*, *HLA-DRB1*, *HLA-DRB2*, *HLA-DRB3*, *HLA-DRB4*, *HLA-DRB5*, *HLA-DRB7*, *HLA-DRB8*) from the International Immunogenetics/HLA database (IMGT) (release 3.31.0 available at https://github.com/ANHIG/IMGTHLA).

For many alleles, sequence data is not available for the entire coding region (e.g., only for exons 2 and 3 for class I, and exon 2 for class II genes, which are called ARS exons). Because the lack of sequences for much of the coding region would cause the exclusion of many reads, including those mapping to the boundaries of the available exons, for each allele with partial sequence we used the available sequence to find the closest allele which has the complete sequence, and attributed the sequence from this allele. This is expected to introduce little bias in either the genotyping step (because ARS exons are the most polymorphic and sufficient to distinguish specific alleles) or the expression estimation (because the non-ARS sequence attributed is likely very similar to the real one).

For the final index file, we replaced the HLA transcripts in the reference transcriptome (Gencode v25, primary assembly) with the HLA diversity described above. STAR’s module genomeGenerate, Salmon’s index, and kallisto’s index compile an index from these sequences.

### *In-silico* HLA typing

In order to select the alleles to be used in the HLA-personalized index, we observed that a simple procedure of selecting the 2 alleles with the largest number of estimated read counts after applying a zygosity threshold is sufficient to produce calls with accuracy of ≥ 95%. However, in order to avoid false homozygotes and false heterozygotes, we implemented additional steps.

First, we selected the top 5 alleles and applied an intra-lineage threshold of 0.25, meaning that only alleles which had at least 25% of the total expression in their lineage were considered for further steps. For each individual, we compiled an index containing only these (up to) 5 alleles and estimated their expression. We then determined if the individual was heterozygote at the lineage level by applying a threshold of 0.15 on lineage expression levels. The lead allele from each lineage was selected to compose the genotype. A zygosity threshold of 0.15 was applied to decide whether the genotype was heterozygous at the allele-level. For each locus, the reads mapped to the lead allele were removed, and another step of alignment and quantification was performed in order to determine if the second allele was real, or just noise due to extensive similarity to the lead allele. If the second allele had at least 1% of the locus read counts, it was kept, otherwise the genotype was considered to be homozygous for the lead allele.

The thresholds described above were chosen because they maximized the concordance with the Sanger sequencing typings [6], while also minimizing the rate of false homozygotes and heterozygotes.

### Expression quantification

We implemented two versions of the HLApers pipeline: (1) one using STAR (v2.5.3a) [34] to map reads followed by Salmon (v0.8.2) [36] to quantify the expression, and (2) using kallisto (v0.43.1) [35], which performs pseudoalignment and quantification.

The quantification pipeline is structured in a two-stage process, first identifying the most expressed allele(s) at each HLA locus in order to infer the genotype which is present, and next quantifying expression for these inferred genotypes as well as for the rest of the transcriptome (Fig 1).

Reads were aligned directly to the transcriptome. STAR alignments were passed to Salmon for quantification (module quant under alignment mode), whereas kallisto directly produces quantifications with the quant module.

In both the HLA typing step (for which the index contains all HLA sequences in the IMGT database) and the quantification step (for which an HLA-personalized index is used), short reads can map to more than one locus, or more commonly to multiple alleles of the same locus (multimaps). The quantification methods we are using deal with multimaps by inferring maximum likelihood estimates optimized by an expectation-maximization algorithm to probabilistically assign reads to each reference in the index, and also include models to account for sequencing bias.

For the mapping with STAR, we tuned parameters in order to avoid discarding multimaps and to accommodate mismatches. For quantification, we used all bias correction options available (–seqBias and –gcBias in Salmon, and –bias in kallisto).

### Simulation

Polyester is an R package designed to simulate RNA-seq datasets [41]. We used the function simulate_experiment_countmat to simulate transcriptome data for 50 randomly chosen GEUVADIS individuals. Simulations were based on the read lengths and counts in the original data, with library sizes of 30 million reads, sampling without bias from a normal distribution of read start sites (with average fragment length of 250bp and sd of 25bp, and error rate of 0.005). The code for the generation of the simulated datasets is available at https://github.com/genevol-usp/hlaexpression/tree/master/simulation/data.

We processed the simulated reads with STAR-Salmon to perform the quantifications using different indices:

1. HLA-personalized index (HLApers): Reference transcriptome with the annotated transcripts for HLA genes replaced with sequences from the personalized genotypes;
2. Reference transcriptome: Gencode v25 transcripts from the primary assembly of reference genome;
3. Reference Genome (GRCh38), considering only uniquely mapped reads.

To investigate the relationship between quantifications and sequence divergence with respect to the reference, we used the R function adist to calculate the proportion of mismatches between the HLA alleles carried by the individuals and the alleles in the reference genome.

### GEUVADIS reanalysis

We quantified HLA expression based on RNA-seq data for 358 European individuals, in samples of LCLs (Lymphoblastoid Cell Lines), made available by the GEUVADIS Consortium [24] (we excluded samples from the original dataset which are not in the 1000 Genomes phase 3).

We performed expression quantification using the HLApers and reference transcriptome pipelines.

We evaluated the reproducibility of the HLA quantifications by analyzing a subset of 97 individuals for which a replicate was available (https://github.com/genevol-usp/hlaexpression/blob/master/geuvadis_reanalysis/replicates/data/write_sample_info.R).

For the eQTL analysis, we used only autosomal genes which are expressed in a large proportion of samples, exploring the thresholds of *TPM* > 0 in at least 25%, 50%, or 75% of samples. In order to correct the expression data for technical effects, we sequentially removed the effect of the first 0 to 100 PCs and ran an eQTL analysis for each condition (S6 Fig). The configuration of thresholds and number of PCs which maximized the eQTL discovery (at FDR = 5%) was considered. This resulted in the use of genes expressed in ≥ 50% of samples (19,613 genes), and 60 PCs.

The PCA analysis and data correction were performed with QTLtools v1.1 [47], using the modules pca and correct respectively. For the genetic variant data, we used the 1000 Genomes Phase 3 biallelic variants, lifted to GRCh38 coordinates, after filtering for *MAF* ≥ 0.05 in the individuals included in this study (6,837,505 variants in total).

In order to control for population structure in the eQTL analysis, we ran a PCA on the variant genotype data and assessed the PCs which captured the structure. We used QTLtools pca requiring that variants should be at least 6kb apart. After visual inspection of the plots in S7 Fig, PCs 1–3 were used as covariates in the eQTL analysis.

We used QTLtools cis to conduct the cis-eQTL analysis using the following model:

PCA-correctedandstandardnormalexpression∼SNPs+covariates(PCsforpopulationstratification)

The permutation pass was performed with 1000 permutations and a cis-window of 1Mb. P-values were computed by beta approximation and significance was determined by running the script runFDR_cis.R provided by QTLtools with FDR of 5%.

Multiple eQTLs with independent effects on a particular gene were mapped with a conditional analysis based on step-wise linear regression (see Supplementary method 8 in [47]). The method automatically learns the number of independent signals per gene and provides sets of candidate eQTLs per signal.

### Functional annotation of eQTLs

In order to investigate the putative function of the eQTLs we mapped, we investigated whether these eQTLs were present in ENCODE [53] regulatory elements annotated for LCLs. We used three types of functional annotations: open chromatin regions given by DNAse footprinting, transcription factor binding sites (TFBS) assayed by ChIP-seq, and histone modifications.

### Regulatory Trait Concordance (RTC) analysis

We performed an RTC [48] analysis as described in [47] to investigate whether our eQTLs tagged the same causal variant as a GWAS variant or previously reported eQTL.

We downloaded the GWAS catalog data (v1.0.1) from https://www.ebi.ac.uk/gwas/api/search/downloads/alternative and selected associations with p-value < 10^−8^. We obtained the coordinates of recombination hotspots from http://jungle.unige.ch/QTLtools_examples/hotspots_b37_hg19.bed.

We applied the RTC module implemented in QTLtools (QTLtools rtc), selecting the HLA region only, using a D’ threshold of 0.5, and turning on the conditional flag (–conditional) to test all independent eQTLs for a gene.

Following the recommendation of Delaneau et al. (see Supplementary Note 7 in [47]), we considered that two SNPs tagged the same functional signal if the RTC score was >0.9.

### Phasing of HLA alleles

To investigate whether there is a haplotypic coordination of expression at HLA, we used phased HLA genotype data to verify if there was more correlation of expression between alleles on the same haplotype than on different haplotypes (Fig 8). To also assess the phasing between HLA alleles and the eQTLs (Fig 6), we included the eQTLs mapped for each HLA gene in the phasing procedure, accounting for the fact that their phasing was already known from 1000 Genomes Project.

In order to be conservative in the phase estimation, we used only the haplotype calls which were concordant between two approaches for phasing. First, we used PHASE [68] to determine the haplotype of each allele in the genotype, providing HLA allele designations and phased eQTL genotypes as input. Second, we checked the compatibility of the individual HLA genotypes with the phased SNP haplotypes from 1000 Genomes. Given all possible haplotypes for each individual at HLA-G∼A∼E∼C∼B∼DRA∼DRB1∼DQA1∼DQB1∼DPA1∼DPB1, we checked which combination of 2 haplotypes minimized the number of differences to the 1000 Genomes data to infer the haplotypes present in the individual. This resulted in 417 haplotypes completely concordant between the two approaches.

### Code availability

The HLApers pipeline is available at https://github.com/genevol-usp/HLApers. The entire analysis, including simulations, index compilation, quantification of expression, eQTL mapping, etc is available at https://github.com/genevol-usp/hlaexpression.

## Supporting information

S1 Fig Gene-level expression of 358 European individuals in the GEUVADIS dataset obtained with the HLA-personalized approach (top panel) or those originally reported by GEUVADIS (bottom). TPM: Transcripts Per Million. FPKM: Fragments Per Kilobase of transcript per Million mapped reads.
(TIFF)

S2 Fig Estimated counts (top) and TPM estimates (bottom) from STAR-Salmon (alignment via suffix array) and kallisto (pseudoalignment). (TIFF)

S3 Fig Expression estimates for replicates of 97 European individuals in GEUVADIS obtained with the HLApers pipeline. Quantification estimates are in TPM (Transcripts per Million). r: Pearson correlation. *ρ*: Spearman correlation.
(TIFF)

S4 Fig Location of HLA genes in cis-regulatory domains (CRD). (TIFF)

S5 Fig CaVEMaN causal probabilities. On the X axis we have the QTLtools rank of the eQTLs, and on the Y axis the CaVEMaN causal probability.
(TIFF)

S6 Fig The number of eGenes (a gene with a least 1 eQTL) according to the number of PCs used for phenotype correction. (TIFF)

S7 Fig Principal components of variant genotypes for 358 European individuals in the GEUVADIS dataset. Genotypes are available from 1000 Genomes. CEU: Utah Residents (CEPH) with Northern and Western European Ancestry. FIN: Finnish in Finland. GBR: British in England and Scotland. TSI: Toscani in Italy.
(TIFF)

S1 Table Accuracy of the *in-silico* HLA genotyping approach. (CSV)

S2 Table RTC analysis between the eQTLs mapped with the HLA-personalized or reference transcriptome pipelines. If two SNPs have an RTC score >0.9 we consider that they share a causal signal.
(CSV)

S3 Table RTC analysis between the eQTLs we mapped and previous eQTLs from studies on LCLs or HLA-target studies. If two SNPs have an RTC score >0.9 we consider that they share a causal signal.
(CSV)

S4 Table Functional annotation of eQTLs from the HLA-supplemented pipeline. (1) Gene, (2) QTLtools rank, (3) rs ID, (4) distance from the gene, (5) presence in transcription factor binding sites (TBFS), (6) DNase I hypersensitive sites (DHS), and (7) histone modifications annotated for LCLs in ENCODE.
(CSV)

S5 Table RTC analysis between the eQTLs mapped with the HLA-personalized pipeline and CRD-QTLs. If two SNPs have an RTC score >0.9 we consider that they share a causal signal.
(CSV)

S6 Table GWAS variants significantly associated with an eQTL in our study (RTC >0.9). (CSV)

S1 Text Comparison between HLA allele-level expression estimates obtained with HLApers and previous qPCR-based estimates. (PDF)
