# Supplementary figures and images for: Expression estimation and eQTL mapping for HLA genes with a personalized pipeline
Source: PLoS Genet. 2019 Apr 22;15(4):e1008091. doi: 10.1371/journal.pgen.1008091 (PMC6497317; doi:10.1371/journal.pgen.1008091)

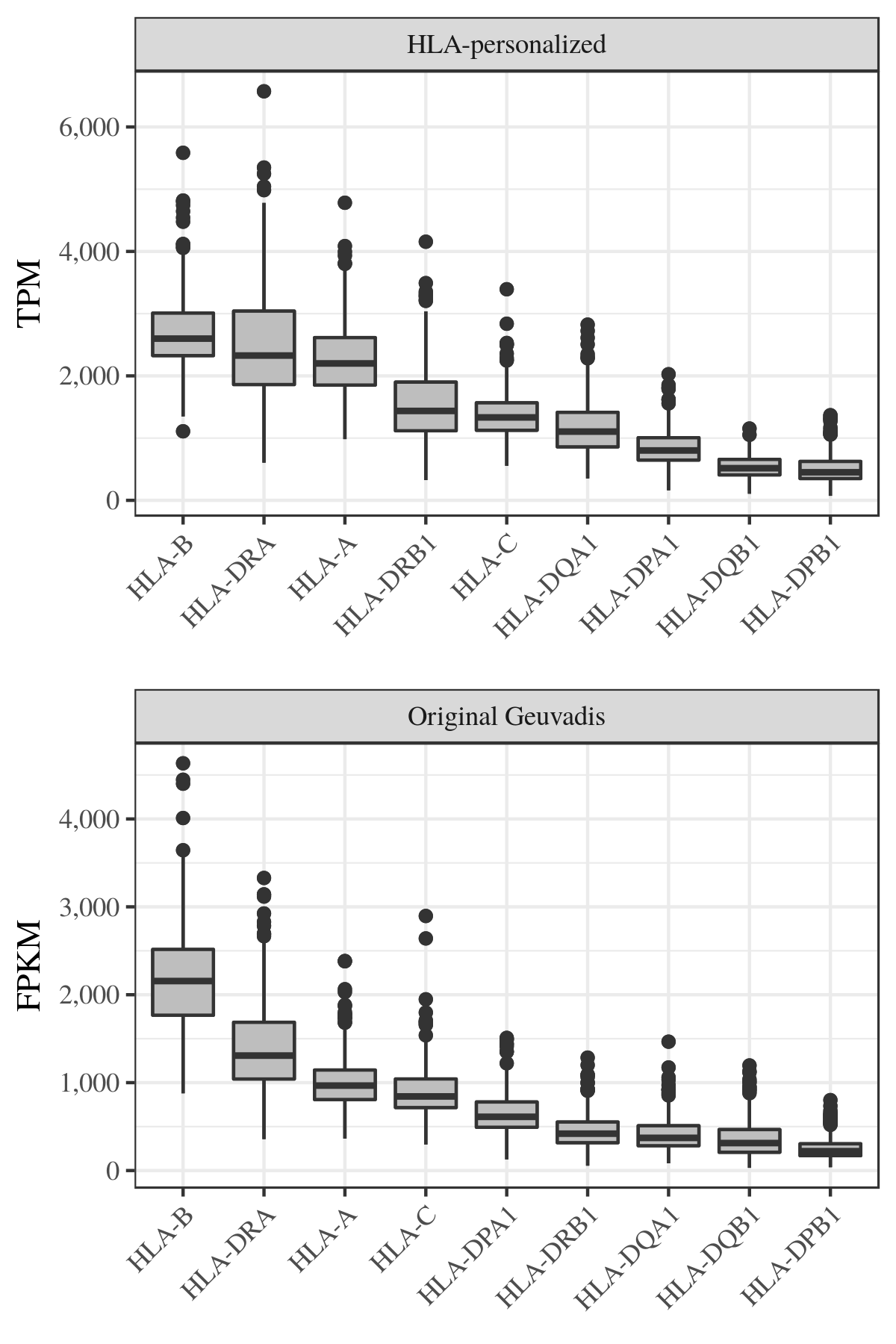

Supplement: S1 Fig — TPM: Transcripts Per Million. FPKM: Fragments Per Kilobase of transcript per Million mapped reads. (TIFF) [file pgen.1008091.s001.tiff]

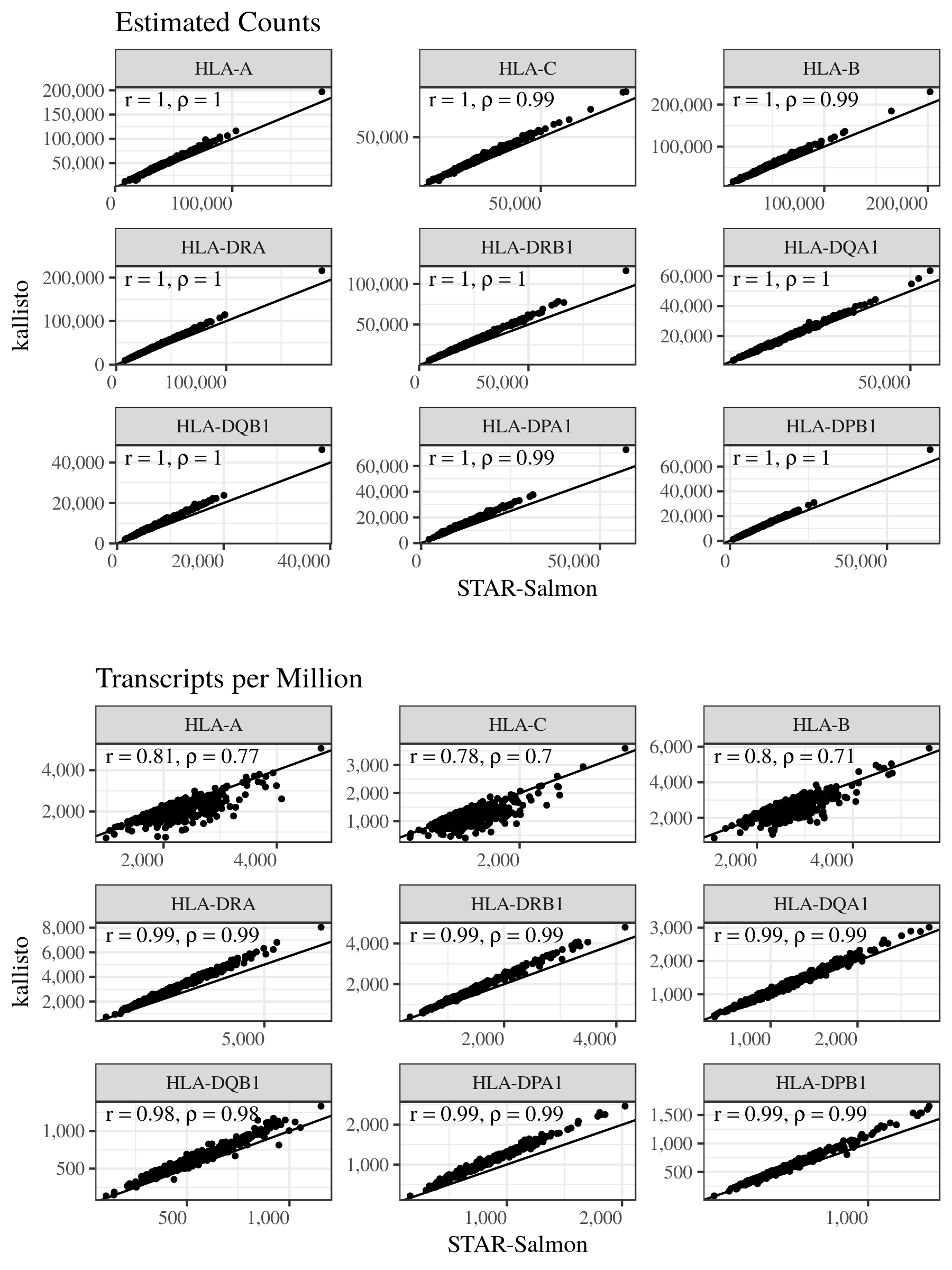

Supplement: S2 Fig — (TIFF) [file pgen.1008091.s002.tiff]

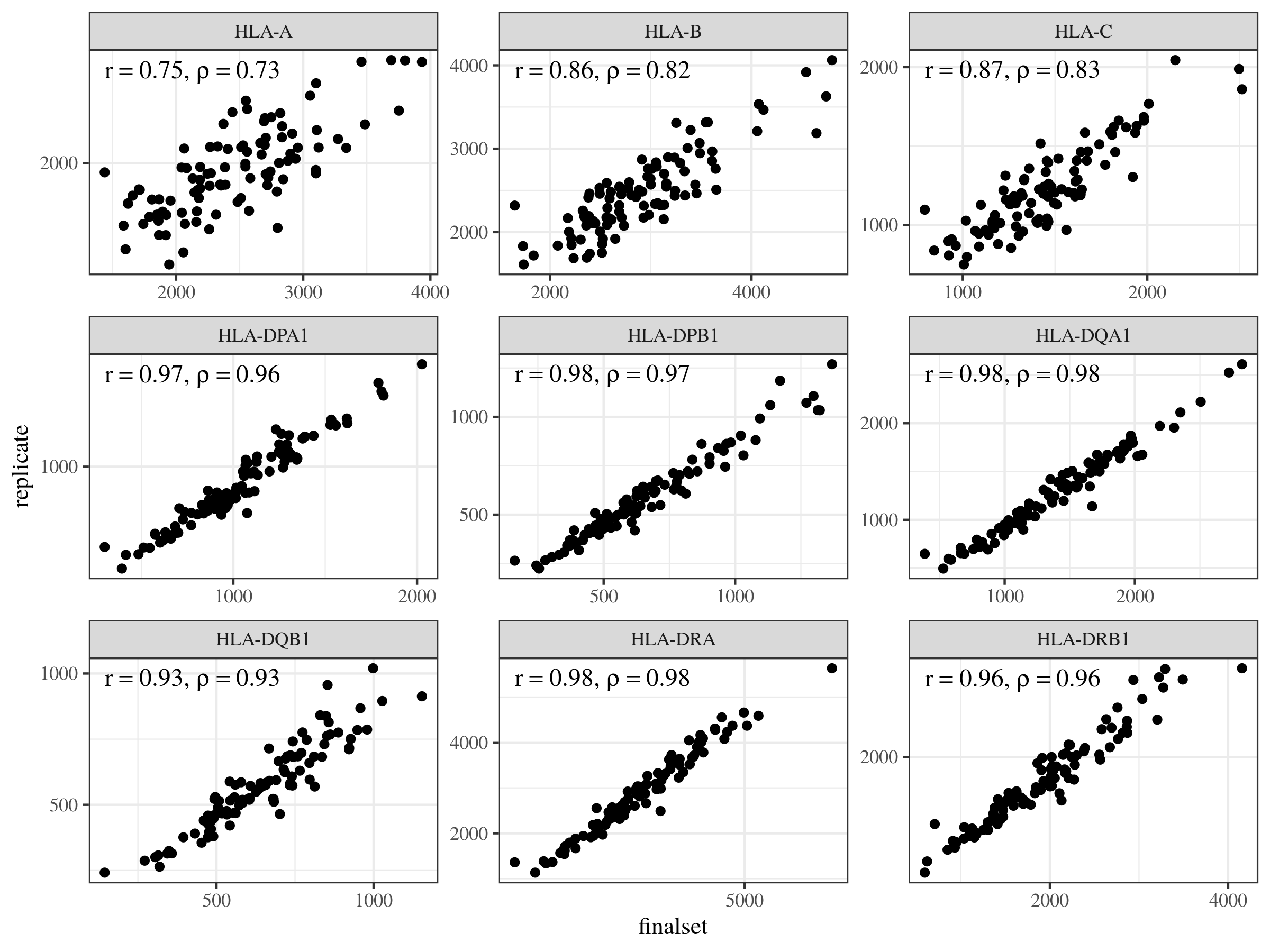

Supplement: S3 Fig — Quantification estimates are in TPM (Transcripts per Million). r: Pearson correlation. ρ: Spearman correlation. (TIFF) [file pgen.1008091.s003.tiff]

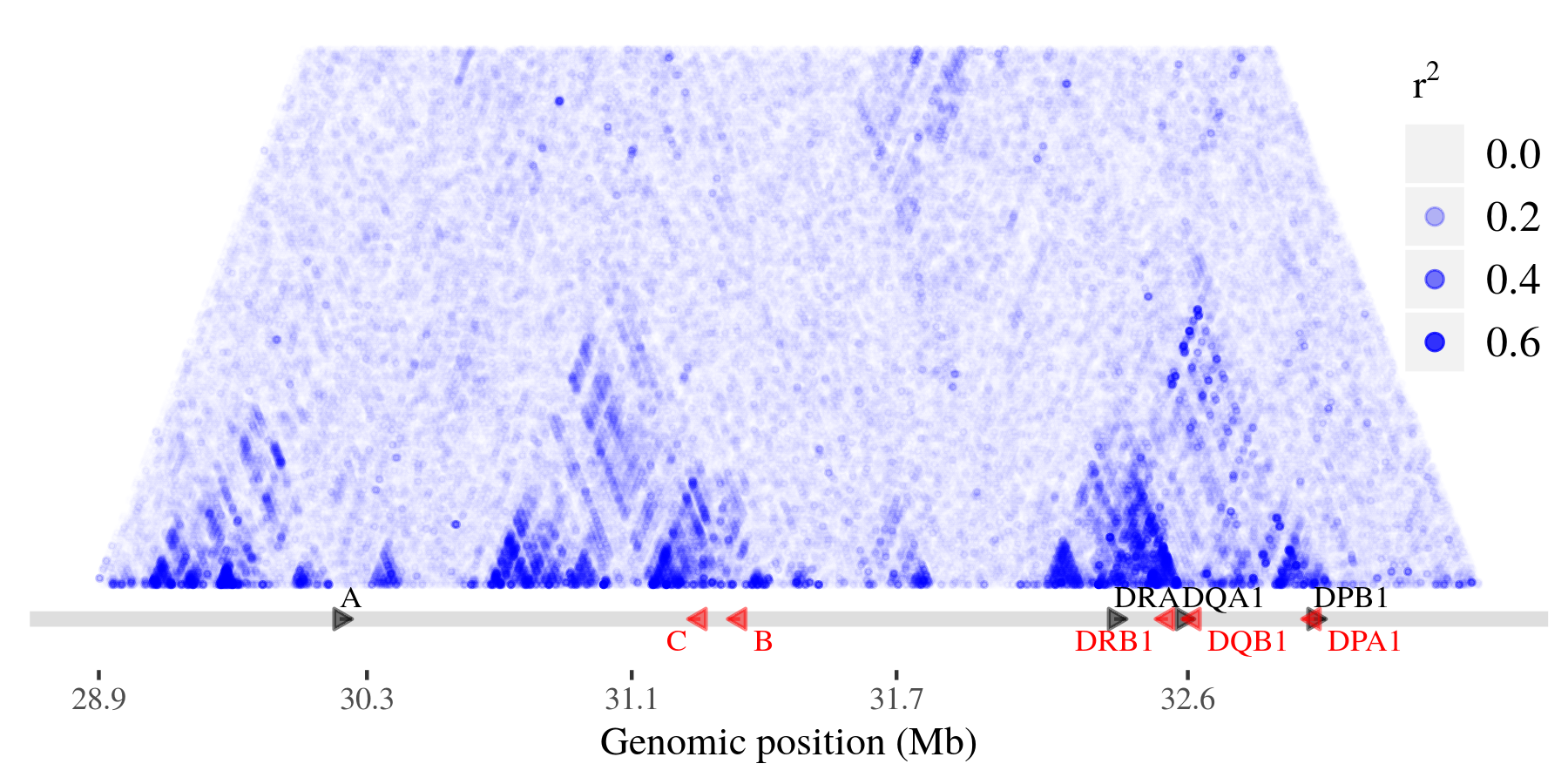

Supplement: S4 Fig — (TIFF) [file pgen.1008091.s004.tiff]

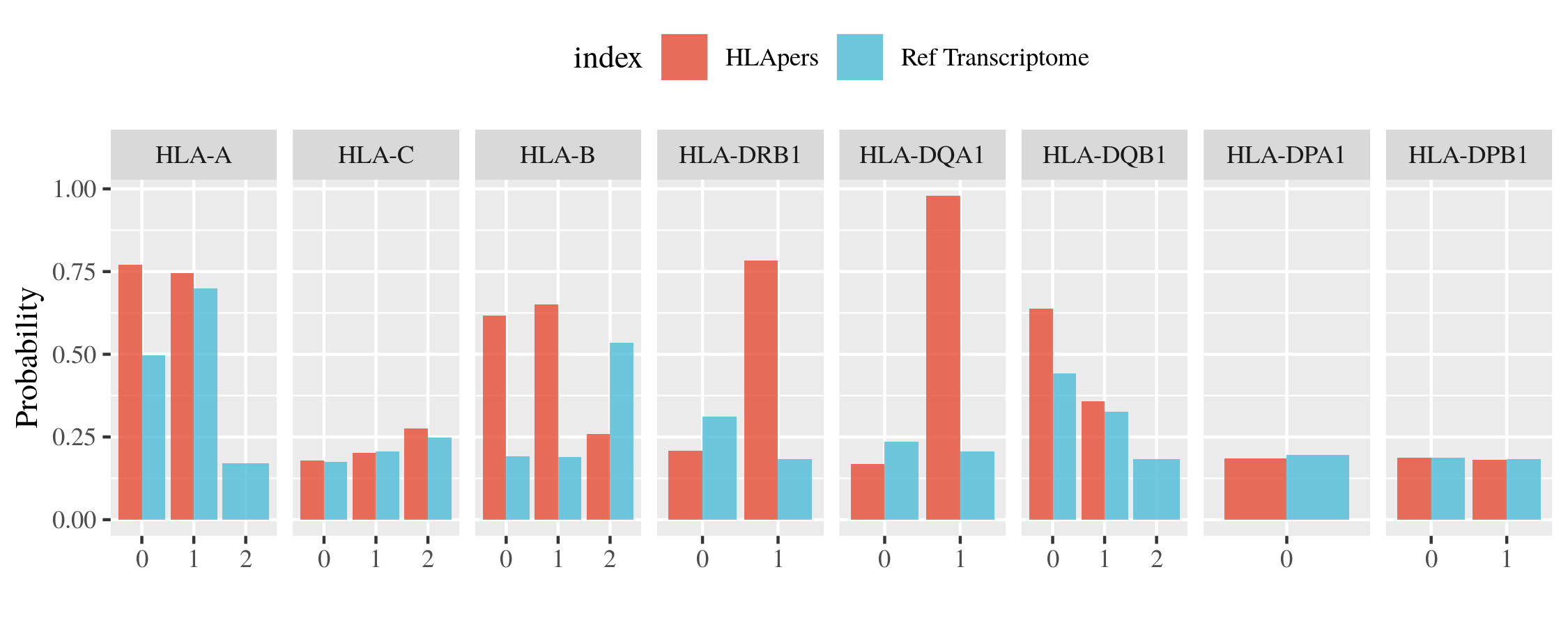

Supplement: S5 Fig — On the X axis we have the QTLtools rank of the eQTLs, and on the Y axis the CaVEMaN causal probability. (TIFF) [file pgen.1008091.s005.tiff]

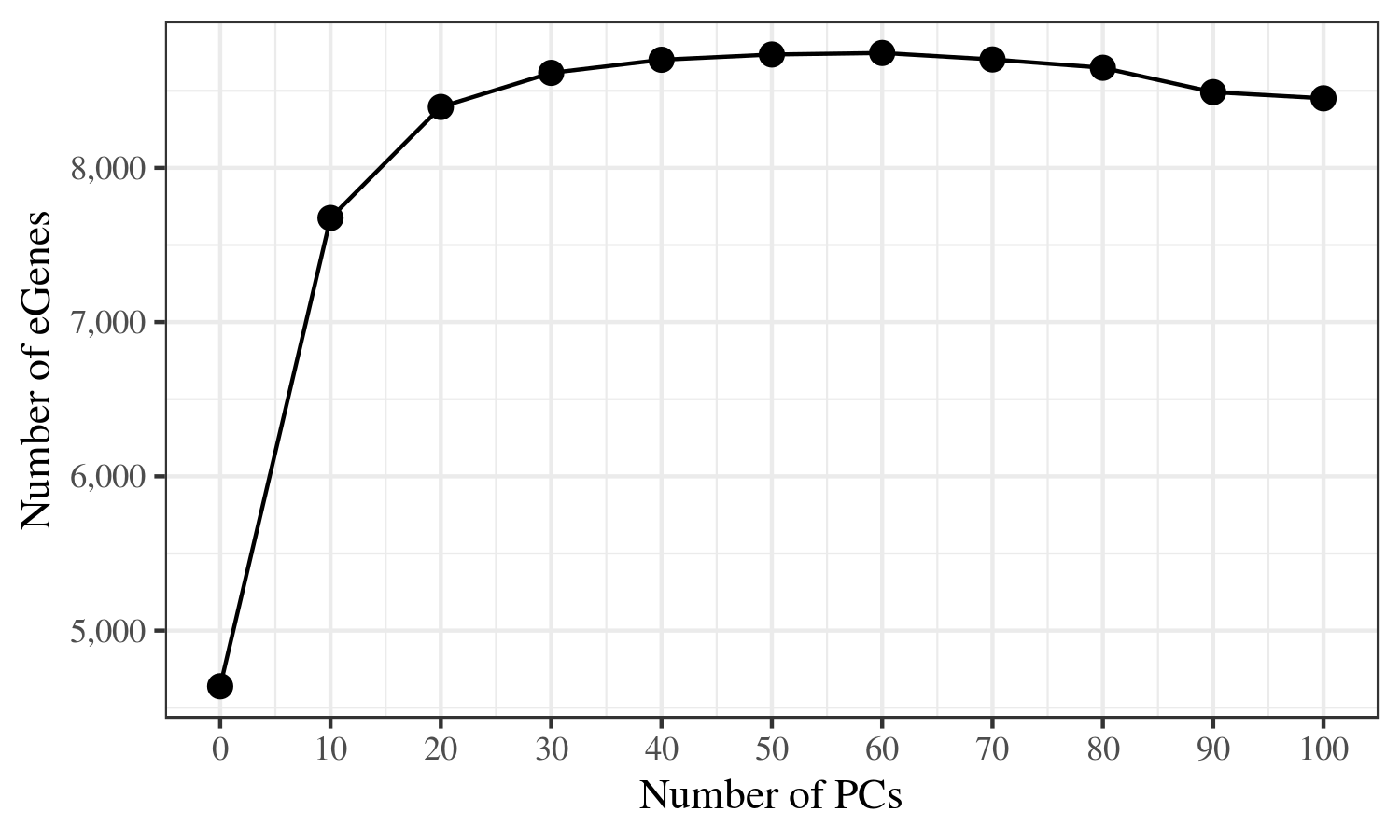

Supplement: S6 Fig — (TIFF) [file pgen.1008091.s006.tiff]

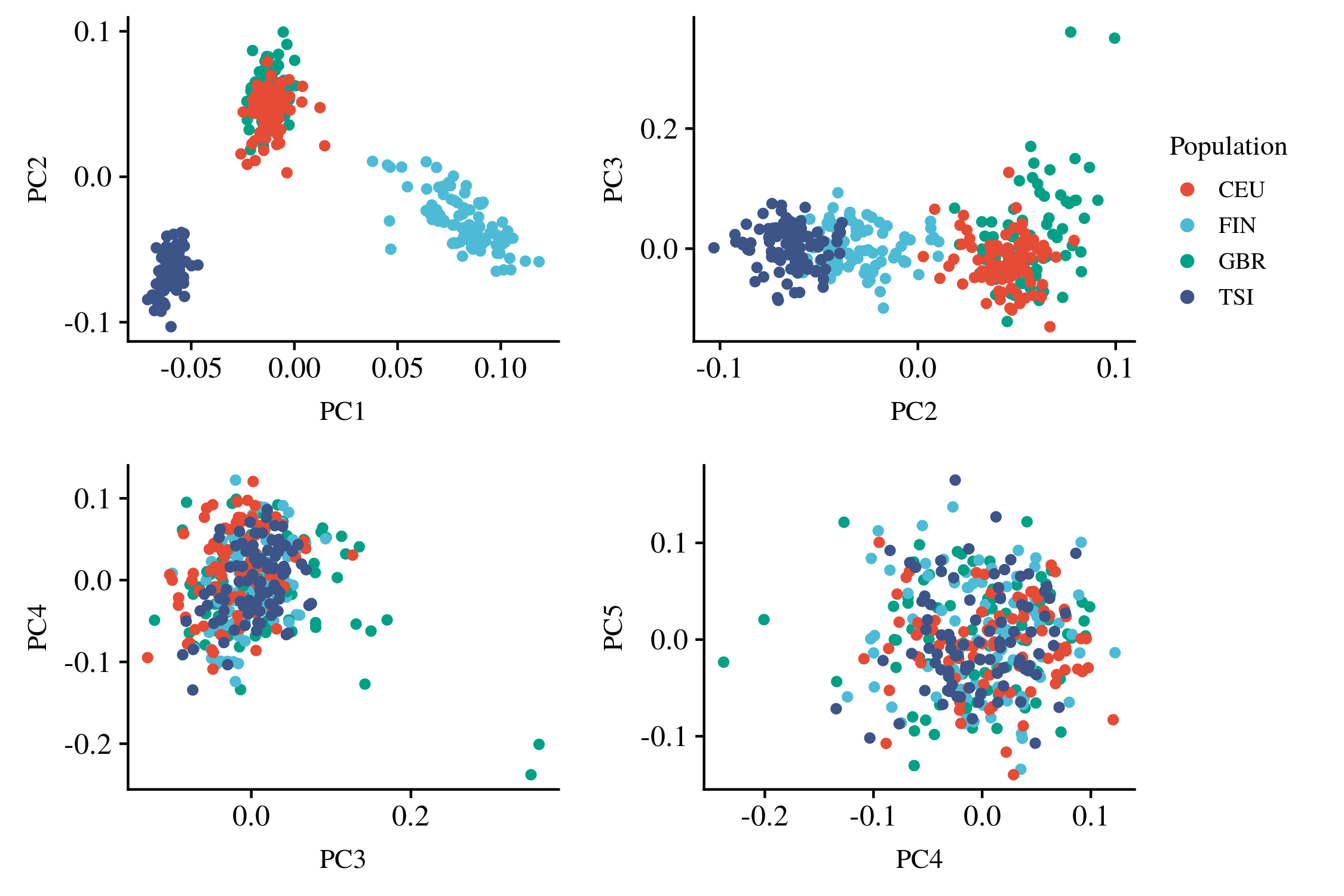

Supplement: S7 Fig — Genotypes are available from 1000 Genomes. CEU: Utah Residents (CEPH) with Northern and Western European Ancestry. FIN: Finnish in Finland. GBR: British in England and Scotland. TSI: Toscani in Italy. (TIFF) [file pgen.1008091.s007.tiff]
