# Supplementary material for: Expression estimation and eQTL mapping for HLA genes with a personalized pipeline
Source: PLoS Genet. 2019 Apr 22;15(4):e1008091. doi: 10.1371/journal.pgen.1008091 (PMC6497317; doi:10.1371/journal.pgen.1008091)
Supplement: S1 Text — (PDF) [file pgen.1008091.s014.pdf]

Supplementary Text 1

Introduction

RNA-seq constitutes a relatively new approach to quantify HLA expression. In order to place our novel findings in the context of well established methods, and because many researchers consider qPCR as a gold standard for HLA expression, we provide an in-depth comparison of RNA-seq with previous qPCR studies.

Of course, our analyses have limitations: when comparing across studies we are often dealing with different individuals, cell types, and techniques. Thus, our efforts to compare our RNA-seq findings to those of published papers that used qPCR are at best a first approximation.

Although some efforts have been made to develop allele-specific primers (Pan et al, 2018), to our knowledge, these have not yet been used to obtain expression levels for population datasets. Thus, allele-based expression levels from qPCR (e.g., Kulkarni et al, 2013; Ramsuram et al, 2015; Ramsuram et al, 2017) are usually imputed from the locus-level expression levels (in the graphs included in those papers, locus-level expression is plotted twice, one point for each allele). Attempts have been made to improve the imputation with the use of a linear model of expression~genotype (e.g., Ramsuram et al, 2015). Thus, besides the technical and biological differences between studies, the imputed nature of allele-level estimates from qPCR provide another source of differences between our RNA-seq estimates with qPCR data at the HLA allele-level. Nevertheless, we designed a survey to compare HLA allele-level expression between RNA-seq and qPCR as rigorously as possible (see below), and to obtain a quantitative summary of the degree of agreement across these methods.

Methods

Data

Expression estimates for *HLA-A*, *HLA-B* and *HLA-C* reported by Ramsuram et al (2015), Ramsuram et al (2017), and Kulkarni et al (2013), were kindly provided by corresponding authors.

Comparison of expression levels between qPCR and RNA-seq

Because our study and the previously published papers with qPCR expression data involve different samples, they cannot be directly compared (e.g., using a correlation analysis). Instead, we asked whether the ordering of HLA lineages by their expression levels differs among studies. To do this, we applied a pairwise Mann-Whitney U test (with FDR correction for multiple testing) to pairwise comparissons between all pairs of lineages with $N \geq 10$ in both the qPCR and RNA-seq data. We restricted subsequent analyses to lineage pairs that showed significantly different expression for both qPCR and RNA-seq quantification.

Results

Comparison of lineage ordering across studies

A first impression of the relative ordering of lineages according to the mean expression level suggests substantial differences between RNA-seq and qPCR quantifications (Figure 1).

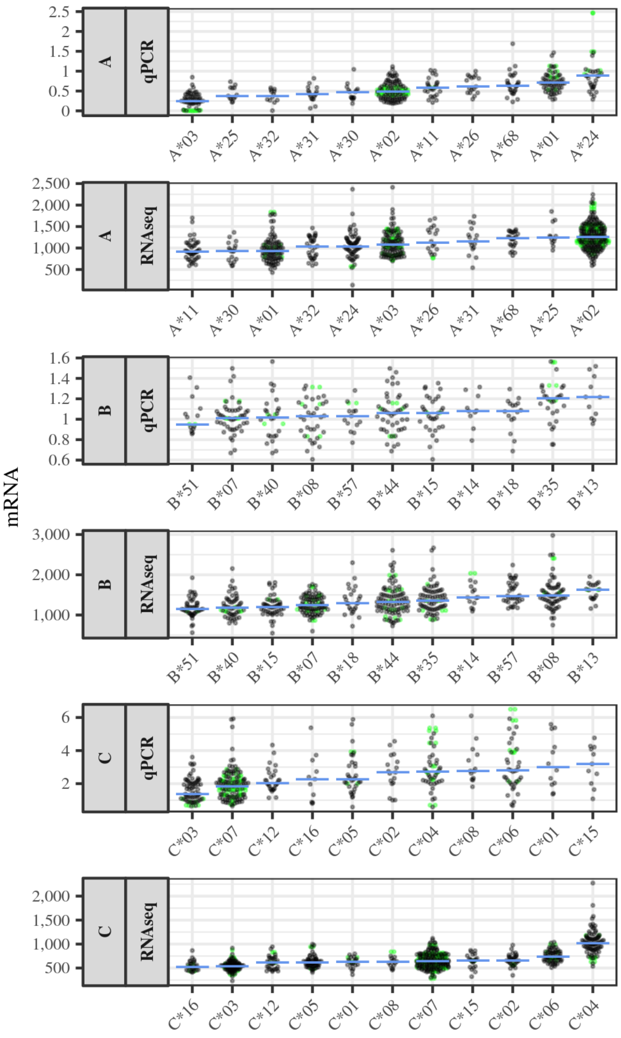

Figure 1. Lineage-level expression in previous qPCR studies and RNA-seq (HLApers) for *HLA-A*, *HLA-B* and *HLA-C*. We included only lineages present in at least 10 individuals in both GEUVADIS RNA-seq data and qPCR data. Y-axis: Transcript per Million for RNA-seq, and $2^{-\Delta\Delta Ct}$ for qPCR. The alleles are ordered from left to right in increasing median expression values.

The apparent lack of agreement among the methods in the ordering of alleles can be misleading. This is because the variation among individuals within the same lineage is often very high, so differences between methods in the ordering of lineages may be within the expected range of sampling variation. We thus restricted our analyses to pairs of lineages with $N \geq 10$, and with statistically different distributions of expression values according to a pairwise Mann-Whitney U test with FDR correction for multiple testing (in both qPCR and RNA-seq).

For *HLA-A*, *HLA-B* and *HLA-C* we found 33 instances where the expression was significantly different for a pair of lineages in both qPCR and RNA-seq datasets (Table 1).

Table 1. Lineage pairs with significantly different expression for both qPCR and RNA-seq (pairwise Mann-Whitney test.

Lineage1	Lineage2	pvalue (pcr)	pvalue (hlapers)
A*01	A*02	0.00000	0.00000
A*01	A*03	0.00000	0.00023
A*01	A*25	0.00028	0.00088
A*01	A*31	0.00004	0.02952
A*02	A*03	0.00000	0.00000
A*02	A*24	0.00000	0.00000
A*03	A*11	0.00000	0.00213
A*03	A*30	0.00134	0.01686
A*11	A*31	0.03687	0.02296
A*24	A*25	0.00017	0.01958
A*24	A*68	0.02551	0.02241
A*26	A*30	0.03268	0.03544
A*30	A*68	0.01782	0.00124
A*32	A*68	0.00236	0.02241
B*07	B*35	0.00180	0.02676
B*08	B*35	0.00995	0.02332
B*15	B*35	0.03133	0.01033
B*35	B*40	0.02262	0.00542
B*35	B*51	0.04514	0.00138
B*35	B*57	0.03133	0.01178
C*01	C*03	0.00083	0.00107
C*02	C*03	0.00085	0.00000
C*03	C*04	0.00000	0.00000
C*03	C*05	0.00003	0.00002
C*03	C*06	0.00000	0.00000
C*03	C*07	0.01296	0.00000
C*03	C*08	0.00002	0.00745
C*03	C*12	0.00187	0.00739
C*03	C*15	0.00112	0.00030
C*04	C*07	0.00000	0.00000
C*04	C*12	0.01816	0.00000
C*06	C*07	0.00000	0.00000
C*06	C*12	0.01296	0.00005

Of the significant pairs, for 21/33 cases the results were concordant (i.e. we found the same ordering of expression in our study and in qPCR-based studies). However, the results were markedly different among loci: for *HLA-C*, 13 out of 13 significant pairs were concordant, for *HLA-B*, 4 out 6 had the same ordering. On the other hand, for *HLA-A*, qPCR and RNA-seq results were concordant in only 4 out 14 pairs (Table 2).

Table 2. Number of lineage pairs concordant between qPCR and RNA-seq, total of significant pairs tested, and percentage of concordance for *HLA-A*, *HLA-B* and *HLA-C*.

Locus	Concordant between qPCR and RNA-seq	Total signif. pairs	Concordance (%)
HLA-A	4	14	28.6
HLA-B	4	6	66.7
HLA-C	13	13	100.0

The high concordance between qPCR and RNA-seq seen for *HLA-C* and *HLA-B* can be exemplified referring to specific lineages: B*35 is consistently more highly expressed than B*07, B*15 and B*40 in both qPCR and RNA-seq, as are C*04 and C*06 in comparison with C*03, C*07 and C*12. The high rate of discordance at *HLA-A*, on the other hand, is driven mainly by the top 2 alleles in qPCR (A*24 and A*01), which have low/moderate expression in RNA-seq, and the top 2 alleles in RNA-seq (A*02 and A*25), which have low/moderate expression in qPCR, and also by A*03, whose homozygotes have expression levels close to zero in qPCR.

The low number of lineages with significantly different expression for *HLA-B* reflects the low variation of expression levels among *HLA-B* lineages in the qPCR data (7 pairs out of 55 were significant, whereas RNA-seq had 31), as well as the low sample sizes of some lineages in the qPCR data. For example, B*13 and B*51 are the most and the least expressed lineages in both qPCR and RNA-seq, but in the qPCR data there are only 11 individuals with B*13 and 14 individuals with B*51, lowering the power of the statistical test. Furthermore, the variability is so high that some individuals with the least expressed lineage (B*51) have higher expression than some individuals with B*13. As a consequence, whereas for RNA-seq these lineages have a significant difference in expression ($p < 4 \times 10^{-6}$), the difference is non-significant for qPCR ($p > 0.07$), excluding the lineage from the subsequent test of concordance.

References

Kulkarni et al. Genetic interplay between HLA-C and MIR148A in HIV control and Crohn disease. PNAS. 2013;110(51).

Pan N et al. Quantification of classical HLA class I mRNA by allele-specific,real-time polymerase chain reaction for most Han individuals. HLA. 2019;91.

Ramsuram V et al. Epigenetic regulation of differential HLA-A allelic expression levels. Human Molecular Genetics. 2015;24(15).

Ramsuram V et al. Sequence and Phylogenetic Analysis of the Untranslated Promoter Regions for HLA Class I Genes. The Journal of Immunology. 2017;198.
